# Initial characterisation of adult human ovarian cell populations isolated by DDX4 expression and aldehyde dehydrogenase activity

**DOI:** 10.1038/s41598-018-25116-1

**Published:** 2018-05-03

**Authors:** Yvonne L. Clarkson, Marie McLaughlin, Martin Waterfall, Cheryl E. Dunlop, Paul A. Skehel, Richard A. Anderson, Evelyn E. Telfer

**Affiliations:** 1 Institute of Cell Biology and the Centre for Integrative Physiology, Hugh Robson Building, University of Edinburgh, Edinburgh, EH8 9XD UK; 2 School of Biological Sciences, Ashworth Laboratories, the King’s Buildings, University of Edinburgh, Edinburgh, EH9 3JR UK; 3 MRC Centre for Reproductive Health, Queens Medical Research Institute, University of Edinburgh, Edinburgh, EH16 4TJ UK

## Abstract

The existence of a population of putative stem cells with germline developmental potential (oogonial stem cells: OSCs) in the adult mammalian ovary has been marked by controversy over isolation methodology and potential for *in-vitro* transformation, particularly where cell sorting has been based on expression of DEAD box polypeptide 4 (DDX4). This study describes a refined tissue dissociation/fluorescence-activated cell sorting (FACS) protocol for the ovaries of adult women which results in increased cell viability and yield of putative OSCs. A FACS technique incorporating dual-detection of DDX4 with aldehyde dehydrogenase 1 (ALDH1) demonstrates the existence of two sub-populations of small DDX4-positive cells (approx. 7 µm diameter) with ALDH1 activity, distinguished by expression of differentially spliced *DDX4* transcripts and of *DAZL*, a major regulator of germ cell differentiation. These may indicate stages of differentiation from a progenitor population and provide a likely explanation for the expression disparities reported previously. These findings provide a robust basis for the further characterisation of these cells, and exploration of their potential physiological roles and therapeutic application.

## Introduction

The question of whether adult female mammals can undergo post-natal germ cell renewal has been debated for almost a century^1^ with the current consensus being that the gamete pool is fixed before or around the time of birth, depending on species^2^. This debate was reignited in 2004 with the publication of a study proposing germ cell renewal in adult mice^3^. Since then the isolation of mitotic cells expressing germline markers has been reported from ovaries of adult rodents^4–9^, cows^10^, sheep^11^ primates^12^ and humans^5,10,11,13–16^. Development of these putative oogonial stem cells (OSCs) *in-vitro*^4–9,11–14,16^ and the generation of live young from fully differentiated rodent putative OSCs^6–9^ has also been described, although no physiological role has been demonstrated in any species.

Scientific opinion however remains divided in regard to the very existence of putative OSCs in ovarian tissue, as well as their developmental potential^17–24^. Critics have argued that experimental techniques, data analysis and interpretation are flawed, and that *in-vitro* transformation could explain the presence of germline markers in mitotically active cells derived from adult mammalian ovaries. Given the controversy surrounding the isolation and definition of these putative OSCs, we sought to avoid the need for *in-vitro* expansion by improving tissue digestion and flow cytometry to sort sufficient adult ovarian cells to allow immediate analysis of gene and protein expression.

Previously, investigators have sorted cells on the basis of detection of the C-terminus of the germline RNA helicase DEAD box polypeptide 4 (DDX4)^5,7,10,12,13,16^. As an adjunct to sorting dissociated cell samples on this basis alone we hypothesised that the activity of a widely recognised marker of viable stem cells, aldehyde dehydrogenase 1 (ALDH1)^25^, would also be present in putative OSCs. We tested this by incorporating ALDH1 activity detection into our FACS protocol, thereby refining our characterisation of the sorted cell populations.

In this study we describe the detection, isolation and analysis of a high number of viable cells sorted from adult human ovarian tissue following a novel manual and mechanical dissociation procedure and high-purity FACS. Analysis of freshly sorted DDX4-positive/ALDH1-positive cells indicated that different subpopulations of DDX4-positive cells could be isolated, distinguishable by expression of distinct *DDX4* transcripts and level of ALDH1 activity, and differential germline gene expression. Preliminary analysis of the ability of DDX4-positive sorted cells to develop into oocyte like structures when combined with somatic cells was also performed.

## Results

### Tissue dissociation

The process of dissociation employed a modified, more manually-based procedure than previously described^5,26^. Extended exposure to enzymes may reduce cell viability^26^ therefore we developed a protocol using repeated, thorough cutting of the adult human ovary tissue prior to mechanical dissociation without intermittent shaking stages^26^, reducing the requirement for enzyme digestion to <2 minutes. This modified method significantly improved both cell survival, determined by using the Trypan blue exclusion viability test (69.4 ± 2.9% viable cells compared to 15.9 ± 3.8% when using published protocols), and post FACS cell yield (0.5–6 × 10^6^ intact cells collected compared to 2 × 10^3^ from 20–100 mm^3^ tissue) when using published protocols^5,26^. Thorough inspection of the dissociated filtrate allows for any remaining oocytes and very small follicles to be removed using a pulled glass pipette prior to antibody incubation thereby preventing primary antibody binding to damaged oocytes reducing the possibility of false positive results.

### Immunocytochemistry and FACS

**Figure 1:**
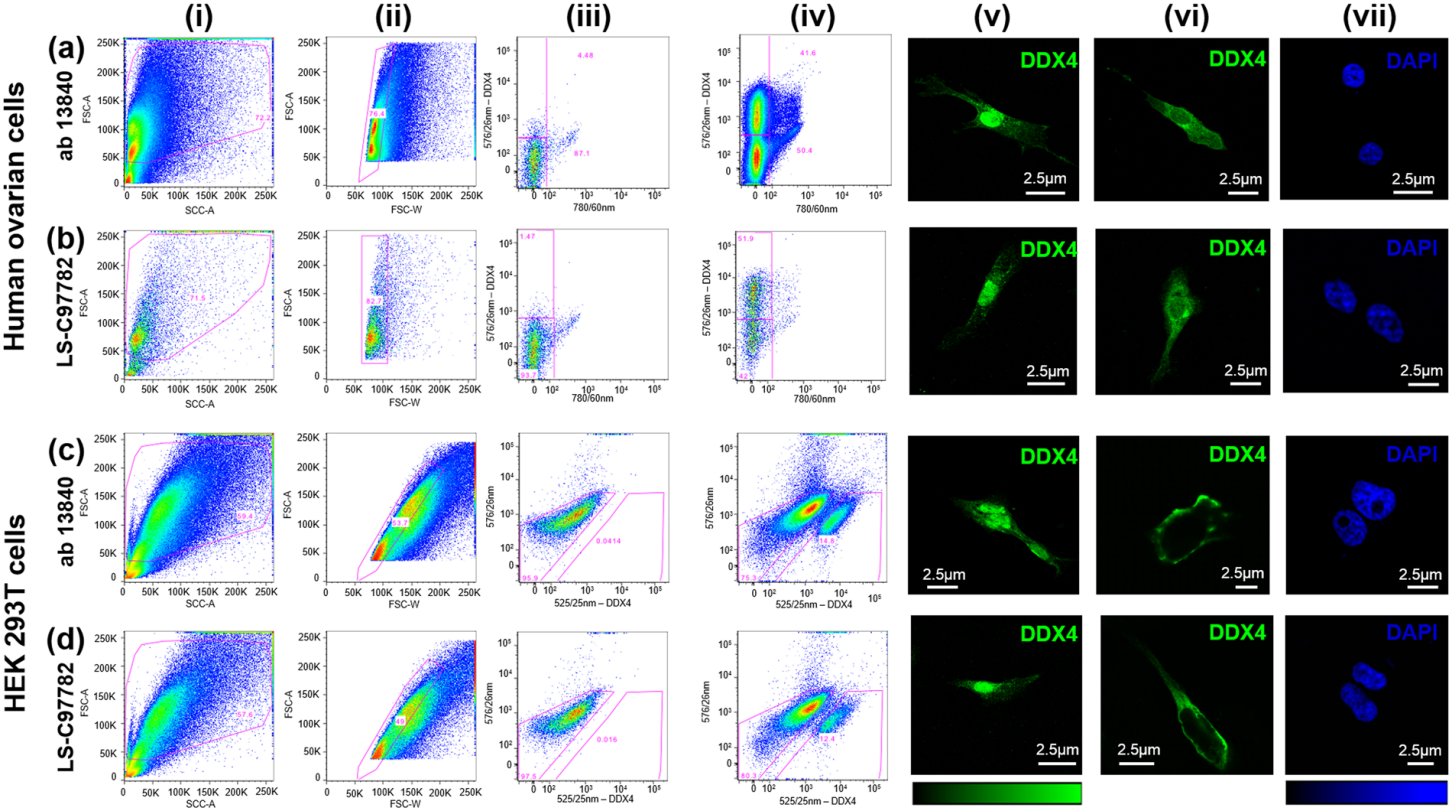
Bivariate flow cytometry plots depicting gating strategies applied to obtain DDX4-positive and negative cells from dissociated adult human ovary and transfected HEK 293T cells. (**a**,**b**) represents sorts from human ovary using ab13840 antibody (**a**) and LS-C97782 antibody (**b**). (i) Sample of dissociated human ovarian cortical cell suspension. Pink line denotes intact cell gate to exclude cell debris and cell fragments based on forward and side scatter profile (72.2% of total sample in (**a**) and 71.5% of total sample in (**b**). (ii) Intact cell aggregates were eliminated by application of a singlets gate on a FSC-A/W plot, pink line denotes intact single cells (76.4% of total intact population in (**a**); 82.7% of total intact population in (**b**). (iii) Negative control, human cell sample with secondary antibody (anti-rabbit Cy3) only added (no primary antibody). DDX4 gating determined by reference to these samples (**a**,**b**). (iv) Staining of DDX4-positive human ovarian cell population positive cells are shown within the upper pink gate. In (**a**) 22.9% of sample detected in the positive gate and in (**b**) 30.7% of sample. A minimum of 20000 cells in total was collected from each gate for further analyses. (**a**,**b**v,vi) represents images of fluorescent immunostained cells sorted using ab13480 antibody (**a**) and LS-C97782 (**b**). Positive DDX4 staining (green) is shown in freshly isolated cells (v,vi) and is located in the nucleus, cytoplasm and cell membrane. No DDX4 staining was detected in negative sorted cells (vii) showing DAPI only in blue. (**c**,**d**i–vii) represent the same type of plots and images as (**a**,**b**) but sorting DDX4 transfected HEK 293T cells where (**c**) has used ab13840 and (**d**) LS-C97782. Gating for DDX4 positive cells is outlined in the pink box on the right of the X axis (**c**,**d**iv). 16.4% of intact singlet transfected cells stained positive for DDX4 in **c** and 13.4% in (**d**), indicating a similar sorting efficiency of both antibodies. Positive sorted HEK 293T cells stained positive for DDX4 (green; **c**,**d**v,vi) but there was no DDX4 staining in the negative sorted cells, stained with DAPI only (blue; **c**,**d**vii). X20 magnification, scale bar, 2.5 µm.

Human ovarian cell suspensions were incubated with a primary polyclonal antibody to sort live cells by DDX4 surface labelling (abcam rabbit anti-DDX4 antibody ab13840). Replicates were carried out using an additional polyclonal anti-DDX4 antibody from a separate supplier (Life Sciences rabbit anti-DDX4 antibody LS-C97782). Viable populations of both DDX4-positive and DDX4-negative single cells were sorted by flow cytometry (n = 10 ab13840 and n = 3 LS-C97782).Tissue was pooled from 3 or more biopsies for each sort with ab13840 (Fig. 1ai–iv) but tissue from only one biopsy was sorted using LS-C97782 (Fig. 1bi–iv). Both antibodies sorted a DDX4-positive and negative population. The proportion of positive cells was similar for both antibodies, ranging from 22.9–30.7%.

To determine whether freshly isolated cells collected from the positive gate were true positives, samples of cells from each sort were immunostained for visualisation. Figure 1av,vi shows images of cells sorted using ab13840 and immunostained after collection and Fig. 1bv,vi shows cells sorted with the LS-C97782 antibody and immunostained with that antibody. Counts of the samples showed that clear positive staining for DDX4 was present in 42.7 ± 2.4% (ab13840) and 38 ± 1.2% (LS-C97782) of cells analysed. DDX4 staining in positive cells was observed within the cytoplasm, nucleus and at the cell surface. Cells collected from the negative population showed no positive DDX4 staining using either antibody ab13840 (Fig. 1avii) or LS-C97782 (Fig. 1bvii).

As a control HEK 293T cells transfected with full length DDX4 (supplementary information, Supplementary Figure S1) were sorted to separate those that had integrated the construct (positive control) and showed surface localisation from those that had not (negative control) using ab13840 (Fig. 1ci–iv) and LS-C97782 (Fig. 1di–iv). The population of positive transfected HEK 293T cells FACS sorted using either antibody was within the range of 13.4–16.4% (Fig. 1c,di–iv) which is indicative of a population with cell surface expression. Fluorescent immunocytochemistry using both DDX4 antibodies was carried out on the positive and negative cells isolated from each group. Positive staining was observed in 52 ± 1.8% (ab13840) and 51 ± 1.2% (LS-C97782) of cells analysed. A similar staining pattern was observed for DDX4 sorted positive cells as was seen with sorted cells from ovarian tissue, examples shown from cells stained with ab13840 (Fig. 1cv,vi) and LS-C97782 (Fig. 1dv,vi). Negative sorted cells showed no staining using either antibody (Fig. 1cvii,dvii).

### Reverse transcription-polymerase chain reaction (RT-PCR) – DDX4 cDNA expression

**Figure 2:**
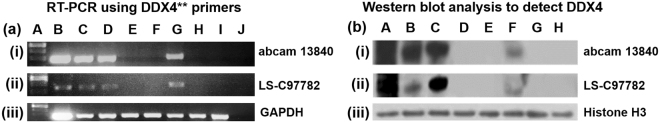
RT-PCR and immuno-blotting of FACS-isolated cell populations. (**a**) RT-PCR of *DDX4* gene expression in human ovarian cells and pFLAG-DDX4-myc transfected HEK 293T cells using both DDX4 antibodies (i, samples FACS sorted using the abcam 13840 antibody and ii, samples FACS sorted using the LS-C97782 antibody). When using primers cited by Anderson *et al*.^27^. *DDX4* expression was detected in dissociated human ovarian cells (i,iiC) and in DDX4-positive sorted human ovarian cells (i,iiD) but not in DDX4-negative sorted human ovarian cells (i,iiE). DDX4 transfected HEK 293T cells sorted in the positive gate showed positive DDX4 expression (i,iiG) but untransfected HEK293T cells (i,iiF) and transfected HEK 293T cells sorted in the negative gate showed no DDX4 expression (i,iiH). Rat skeletal muscle was used as a negative control and showed no gene expression (i,iiI). Reverse transcriptase was omitted from duplicate reactions as negative controls (i,iiJ); internal control: GAPDH (iii); A, ladder, B, template DNA complimentry to DDX4 primers pairs. **primer sequence from Anderson *et al*.^27^. Samples were derived from the same experiment and gels were processed in parallel. Full-length gels can be seen in Supplementary Figure S2. (**b**) Western blot analysis of DDX4 protein by two different DDX4 antibodies (i, samples immunoblotted using the abcam 13840 antibody and ii, samples immunoblotted using the LS-C97782 antibody) in sorted adult human ovarian cells and transfected HEK 293T cells. DDX4 was expressed in rodent testes (i,iiA), dissociated human adult ovary (i,iiB), DDX4-positive sorted human adult ovarian cells (i,iiC) and DDX4-positive sorted HEK 293T transfected cells (i,iiF). DDX4 was absent from human adult ovarian DDX4-negative sorted cells (i,iiD), untransfected HEK 293T cells (i,iiE), DDX4 negative sorted HEK 293T transfected cells (i,iiG) and rat skeletal muscle (i,iiH; which was used as a negative control); Histone H3 was used as an internal protein loading control. Full-length blots are presented in Supplementary Figure S3.

Cellular *DDX4* gene expression was investigated in the DDX4-positive and negative sorted cell populations from adult human ovary sorted using both DDX4 antibodies, ab13840 (n = 4 Fig. 2ai) and LS-C97782 (n = 3 Fig. 2aii). Positive controls for DDX4 were dissociated adult human ovary (Fig. 2aC) and positive sorted DDX4 transfected HEK 293T cells (Fig. 2aG). Negative controls included untransfected HEK 293T cells (Fig. 2aF), negative sorted transfected HEK 293T cells (Fig. 2aH) and rodent skeletal muscle (Fig. 2aI). 2 sets of *DDX4* primers were used; the first of which was reported previously by White *et al*.^5^ for analysis of putative OSCs, and the other by Anderson *et al*.^27^ for analysis of human fetal ovary. Primers described by White *et al*.^5^ failed to detect any *DDX4* gene expression in any of the tested samples. However, using the primers reported by Anderson *et al*.^27^, *DDX4* expression was consistently detected in dissociated adult human ovarian tissue (Fig. 2aC), in DDX4-positive sorted human ovarian cells (Fig. 2aD) and in DDX4-positive sorted HEK 293T transfected cells (Fig. 2aG), after FACS sorting with either antibody (Fig. 2ai,ii and Supplementary Figure S2). Expression was not detected in DDX4-negative sorted human ovarian cells (Fig. 2aE), untransfected HEK 293T cells (Fig. 2aF), DDX4-negative sorted HEK 293T transfected cells (Fig. 2aH) or sorted rodent skeletal muscle cells (Fig. 2aI) using this primer set Fig. (2ai,ii). GAPDH was used as a loading control (Fig. 2aiii).

### Protein detection by Western blot analysis

DDX4-positive and negative sorted cells, from both adult human ovary and transfected HEK 293T cells, sorted using the ab13840 antibody, were lysed and protein samples were generated. DDX4 protein was detected in both DDX4-positive sorted cell populations (Fig. 2bC and F) using either the ab13840 (Figs 2bi and S3) or LS-C97782 (Figs 2bii and S3) antibodies. DDX4 protein was not detected in any of the protein extracts from cells shown to be DDX4 negative by sorting (Fig. 2bD and G). Positive controls were established using rodent testes (Fig. 2bA) and dissociated adult human ovary (Fig. 2bB); negative controls were established using untransfected HEK 293T cells (Fig. 2bE) and rodent skeletal muscle (Figure 2bH). Histone H3 was used as an internal loading control (Figs 2biii and S3)

### FACS by combined DDX4/ALDH1 activity

**Figure 3:**
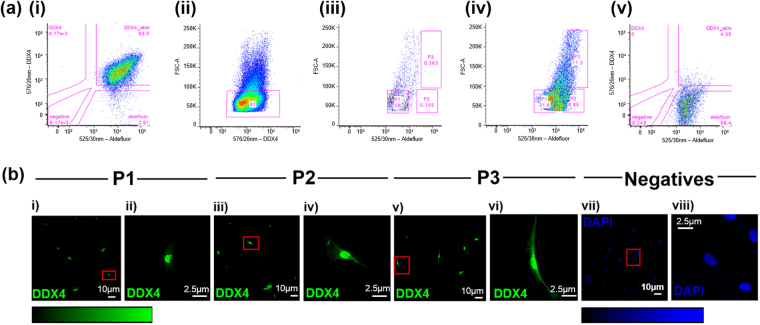
(**a**) Adult human ovarian and rat skeletal cells dual labelled with DDX4 and Aldefluor. Pink lines and boxes denote gates as indicated. Sorting and analysis utilise the same FSC, SSC and singlet gating strategy as previous sort. (i) Human ovarian cells dual labelled with DDX4 (em.576/26 nm) and Aldefluor (em.525/30 nm–93.5% of the total DDX4-positive sample). (ii) The size distribution of DDX4-positive cells indicate >80% have a mean diameter of ~8 µm. (iii) DDX4-positive sample stained with Aldefluor and quenched by DEAB. Sort gates applied to DDX4-positive cells with reference to cell size (FSC) and Aldefluor intensity. (iv) Sub-populations of sorted human ovarian cells (P1, P2 and P3) dual-labelled with DDX4 and Aldefluor. P1 and P2 represent the smallest cells with the P2 population demonstrating higher ALDH1 activity than P1 (8.99% of total DDX4-positive Aldefluor positive cells). (vi) Rat muscle cells was used as a negative control for DDX4 and to show a single emission profile of Aldefluor (em.525/30 nm–88.4% of the total sample). (**b**) DDX4 immunostaining of the DDX4 positive, P1, P2 and P3, and negative sorted cells. Positive staining (green) is observed in each population of DDX4 positive sorted cells P1 (i,ii), P2 (iii,iv) and P3 (v,vi). There was no DDX4 fluorescent signal detected in the negative cells (vii and viii). DAPI was used to stain the nuclei of these cells (blue) for visualisation. Linear LUTs present underneath the images. X20 magnification, scale bar, 10 µm (zoomed image, 2.5 µm).

To refine further the phenotype and identification of putative OSCs, human ovarian cell suspensions were co-labelled with Aldefluor and DDX4 (ab13840). Cells were sorted based on dual-expression for DDX4 and ALDH1 activity (*n* = 4; Fig. 3ai–iv). Three sub-populations of DDX4-ALDH1- positive cells were defined and sort gates applied based on forward scatter (FSC) and ALDH1 intensity: FSC^low^ALDH1^low^ (Population 1; P1), FSC^low^ALDH1^high^ (Population 2; P2) and FSC^high^ALDH1^high^ (Population 3; P3). The diameter of cells collected in each population was measured by reference to beads for size calibration. Cell sizes in P1 and P2 ranged from 3–10 µm whilst P3 cells were between 11–18 µm. Rat skeletal muscle was used as a DDX4 negative ALDH-1 positive control^28^ (Fig. 3av). On re-acquisition of sorted DDX4-ALDH1-positive cells (1000 intact events), ≥ 93% of cells fell within expected gating criteria.

After sorting, DDX4-negative and DDX4-positive (P1, P2 and P3) were immunostained using ab 13840. Fluorescent signal was detected in DDX4-positive cells (Fig. 3bi–vi), but no DDX4 signal was detected in the DDX4-negative sorted cells (Fig. 3bvii,viii). The nucleus of these cells was stained with DAPI for visualisation.

### RT-PCR: DDX4 cDNA expression in ALDH1 positive cells

**Figure 4:**
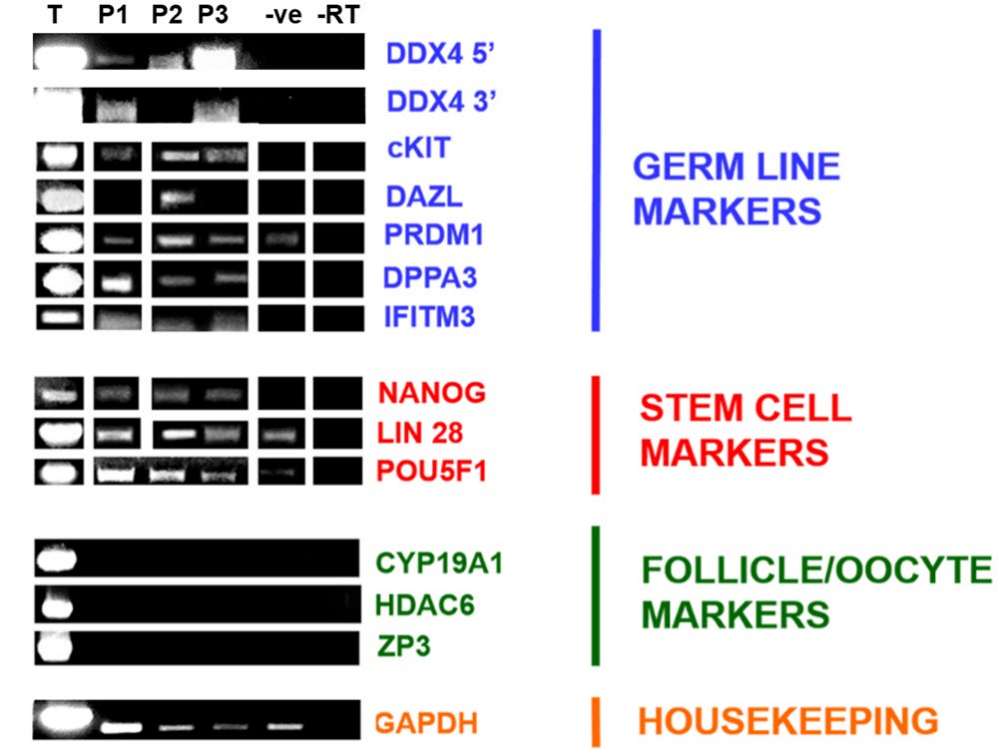
RT-PCR of stem cell and germline markers in DDX4 positive and negative sorted cells. Template DNA complimentary to primers was used as a positive control (T). Expression analysis of germline (blue -DDX4 5′ and 3′, cKIT, DAZL, PRDM1, DPPA3 and IFITM3), stem (red-NANOG, LIN28 and POU5F1) and oocyte markers (green-CYP19A1, HDAC6 and ZP3) in sub-populations (P1, P2 and P3) of sorted human ovarian DDX4-positive and DDX4-negative cells (−ve). GAPDH was an internal control (brown). Reverse transcriptase was omitted from duplicate reactions as negative controls (−RT). Samples were derived from the same experiment and gels were processed in parallel. White spaces between bands indicate when 1) samples were not run adjacently on the same gel, 2) bands have been grouped from different gels and 3) different exposures have been used for clarity. Full-length gels can be seen in Supplementary Figures S5.1–5.7.

Primers specific for 5′ and 3′ regions of the *DDX4* transcript (Table 1 and Supplementary Figure S4) were used on cDNA derived from RNA isolated from the DDX4-positive cells sorted on the basis of size and ALDH1 activity. All sub-populations of DDX4-positive cells expressed transcripts containing the 5′ *DDX4* sequence whereas the 3′-specific primers detected transcripts in P1 and P3 only, indicating that the P2 population expresses a splice variant of *DDX4* with a distinct exon splicing pattern (Fig. 4). DDX4-positive cells in P1, P2 and P3 expressed the pluripotency markers *POU5F1*, *LIN28* and *NANOG* and the germline markers *cKIT*, *DPPA3*, *IFITM3* and *PRDM1*. *DAZL* expression was only found in DDX4-positive cells in P2, which did not express *DDX4* transcripts containing the 3′-specific PCR priming sites. No expression of oocyte/follicle markers (*CYP19A1 (*also known as *Aromatase)*, *HDAC6* or *ZP3*) was detected in any DDX4-positive cell population (Fig. 4 and S5.1–S5.7). DDX4-negative cells did not express *DDX4* (using either set of primers) or any oocyte markers but did express the stem cell markers *POU5F1* and *LIN28* and germline marker *PRDM1*.

**Table 1:** Details of Primer Sequences.

Gene	Accession No.	Forward 5′-3′	Reverse 5′-3′	Size (bp)	Template No.	Template Size (bp)	Annealing Temp (°C)
DDX4*	NM_024415	TTGTTGCTGTTGGACAAGTGGGTG	GCAACAAGAACTGGGCACTTTCCA	283	3	345	59
DDX4**	NM_024415	AAGAGAGGCGGCTATCGAGATGGA	CGTTCACTTCCACTGCCACTTCTG	239	3	297	59
*NANOG*	NM_001297698	TGGAGCAACCAGACCCAGAACATC	AGGAAGGATTCAGCCAGTGTCC	360	2	450	59
*Lin 28*	NM_024674	AGTCAGCCAAGGGTCTGGAATC	GGGAAGAAAGGGTGATGGTGTG	480	3	344	59
*POU5F1*	NM_001173531	GAAAGAGAAAGCGAACCAGTATCG	GCAGGCACCTCAGTTTGAATGC	404	3	340	55
*DDX4 5*′	NM_024415	AGTCAGAAGCAGAAGGAGGAGAAAG	TGGTAGGAGAAAAGCCGCAGTC	353	3	337	55
*DDX4 3*′	NM_024415	GCTCTTGGAGATTTCGCTTTGG	TGCTCTTGCCCTTTCTGGTATC	364	3	339	55
*KIT*	NM_000222	AGAGCAAATCCATCCCCACAC	TTGAGCATCTTTACAGCGACAGTC	339	3	336	59
*DAZL*	NM_001190811	ATCTTTGCCGCACGAGTCTACTGC	CATTCTGAAACTGTGGTGGAGGAG	437	3	345	55
*PRDM1*	NM_001198	GGAAGGCTTTACCAACCTGTCTC	CCCGCTTTTACCCCAAGATG	408	3	341	55
*DPPA3*	NM_199286	TTGAGGCTCTGTCATCAGTTTCTG	CCTTAGGCTCCTTGTTTGTTGGTC	433	3	345	55
*IFITM3*	NM_021034	TGTCGTCTGGTCCCTGTTCAAC	GCCATTGTAGAAAAGCGTGTGAG	367	3	339	60
*CYP19A1*	NM_000103	CACACCAGAGAACCAGGCTACAAG	TGAATGTTGCTTTTCCACCTCC	326	2	440	56
*HDAC6*	NM_006044	ATCACGCCCAGCACAGTCTTATGG	TGAATGTTGCTTTTCCACCTCC	330	2	450	60
*ZP3*	NM_001110354	ATGTGGTCAGGTTTGAGGTTGG	TCCATCAGACGCGAGAGAAAGTC	271	2	447	60
*GAPDH*	NM_001256799	AAGGTGAAGGTCGGAGTCAACG	TGGAAGATGGTGATGGGATTTC	227	3	335	55

^*^Primer sequence from White *et al*.^5^.

^**^Primer sequence from Anderson v*et al*.^27^.

### *In-vitro* potential of DDX4-sorted cells

**Figure 5:**
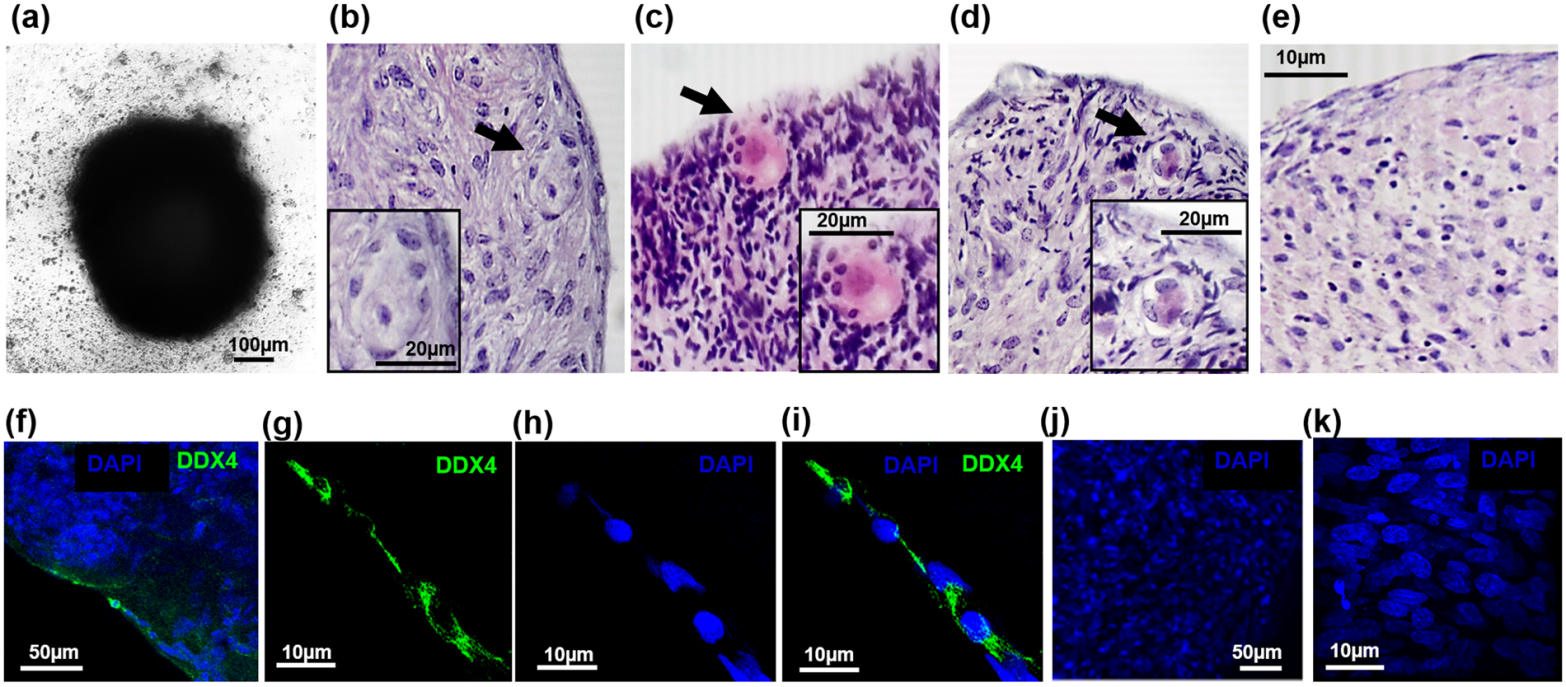
Images of aggregates formed from DDX4-positive cells and fetal ovarian derived somatic cells (FODSCs). Defined aggregates are formed within 10 days (**a**). Histological sections of the aggregates stained with Haematoxylin and Eosin (H&E) show the formation of follicle like structures (**b**–**d**). These comprised of a large oocyte-like eosin stained cell with a nuclear structure surrounded by somatic cells (inserts **b**–**d**). 1–3 follicle like structures were observed per aggregate in 57% of aggregates where they were found close to the edges (**b**–**d**). No structures were observed in any aggregates comprised of FODSCs alone (**e**). x20 magnification, scale bars, 100 µm, 20 µm and 10 µm. Confocal imaging showed the presence of DDX4-positive cells (green) within aggregates (**f**–**i**) when immunostained for DDX4 using ab13840. Low power magnification (X20) (**f**) shows staining for DDX4 (green) close to the edges of the aggregates with surrounding cells staining positive for DAPI (blue) only. High power magnification (X63; **g**–**i**) of the DDX4-positive stained cells shows elongated cells with DDX4 staining predominantly in the cytoplasm and cell membrane (green, **g**) with the nucleus staining positive for DAPI (**h**); merged images (**i**). No DDX4-positive cells were observed in aggregates comprised of FODSCs alone (**j**,**k** DAPI staining (blue) used for cellular visualisation). X20 magnification, scale bar 50 µm. x63 magnification, scale bar 10 µm.

To investigate the developmental potential of FACS sorted DDX4-positive cells isolated from human ovarian cortex, aggregates of cells comprising of DDX4-positive sorted cells and human fetal ovarian derived somatic cells (FODSCs) were generated *in vitro* (n = 14). Control aggregates comprised of FODSCs only (n = 5). After 10 days in culture defined aggregations could be observed (Fig. 5a). Histological analysis detected small putative follicle like structures (diameter 10–20 µm) in 8 of 14 DDX4-positive cell/FODSC aggregates (Fig. 5b–d). When putative follicle structures were observed within an aggregate their frequency was 1–3 per aggregate. These structures comprised of a single oocyte like structure with eosin stained cytoplasm surrounded by somatic cells (Fig. 5b–d inserts). No putative follicle structures were observed in aggregates containing FODSCs only (Fig. 5e).

Confocal microscopy was used to analyse aggregates that had been cultured for 10 days, and immunostained for DDX4. DDX4 positive cells were observed within the aggregates composed of DDX4-positive sorted cells and FODSCs, and could be observed at the edge of the aggregates (Fig. 5f–i). No cells stained positive for DDX4 in the FODSCs alone (Fig. 5j,k).

**Figure 6:**
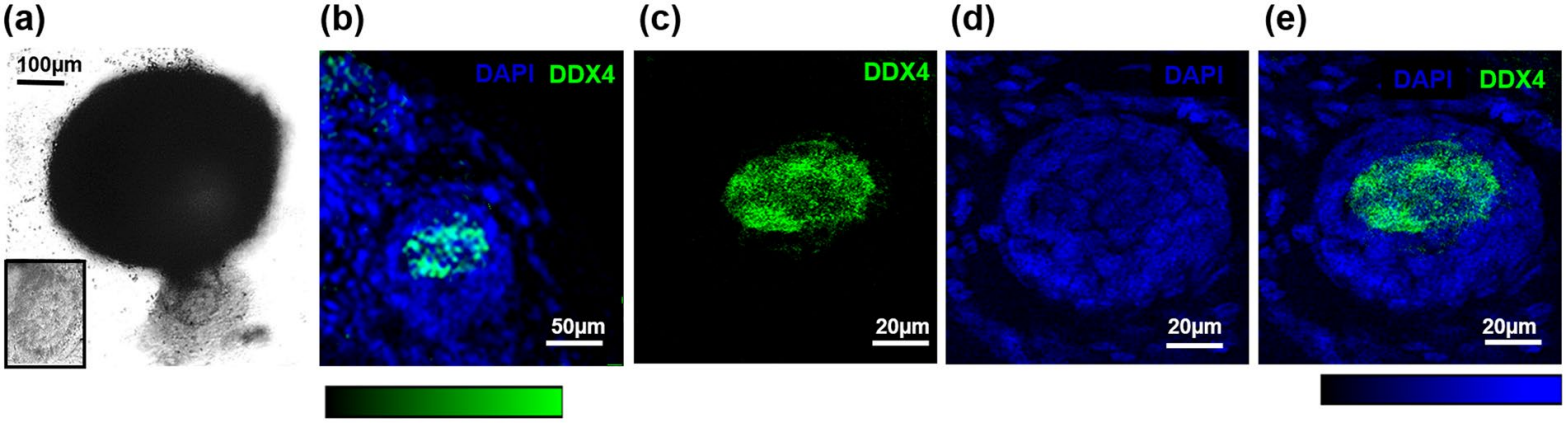
(**a**) DDX4-positive structure was emitted from an aggregate comprised of DDX4-positive sorted cells and FODSCs after 10 days *in vitro*. Bright field microscopy (x20 magnification, scale bar 100 µm) identified a structure emitted from an aggregate (**a**). Confocal images of the emitted structure fluorescently immunostained for DDX4 (green) and DAPI (blue) (**b**–**e**). A DDX4-positive structure is observed within surrounding DDX4-negative cells at X20 magnification, scale bar 50 µm (**b**). The DDX4 structure at X63 magnification measures 40 µm (**c**). DAPI staining confirms a multi-laminar structure measuring 60 µm at X63 magnification (**d**). A merged image shows a multi laminar follicle like structure containing a DDX4-positive central structure, X63 magnification, scale bar 20 µm (**e**).

Interestingly, structures were noted that appeared to be in the process of emission from the aggregates (Fig. 6a). Confocal analysis of one such structure revealed an organised “mini-aggregate” that was comprised of a multi laminar structure embedded within somatic cells. This structure resembled a multi laminar follicle (Fig. 6b), with somatic layers at the edges and a DDX4 positive structure of 40 µm diameter within the centre (Fig. 6b–e). These were only observed in combined aggregates and not in aggregates containing somatic cells alone.

## Discussion

Isolation and development of putative ovarian stem cells from post-natal rodent, monkey, pig and human tissue has been reported by several groups^4–9,12–15,26^, with replacement of these cells in infertile animals resulting in live birth in rodents^6,7,9^ and formation of follicle/oocyte like structures in other species. The premise of these studies challenges the accepted dogma, held for decades, of a non-renewable fixed ovarian pool^2^. There still remains little evidence to support neo-oogenesis under physiological conditions but there is an increasing body of work supporting the presence of cells within adult ovaries that may be oogonial stem cells (OSCs).

Critics^18,20^ highlight reliance on techniques to detect a putative surface epitope of the RNA helicase DDX4 to isolate putative OSCs. Some commentators have considered DDX4 to be an intracellular protein^29^. DDX4 is expressed in the cytoplasm of oocytes, however, DDX4 protein is found in a range of tissues and cells where its subcellular localisation can be nuclear, cytoplasmic or membrane bound, with potentially dynamic localisation depending upon cellular context^29^. The present data provide evidence that populations of cells expressing DDX4 can be isolated from adult human ovary and can be analysed without the need for *in-vitro* expansion. The isolated cells are not homogeneous, possibly reflecting variation in differentiation status, and can, with an appropriate somatic cell environment, form follicle-like structures.

To isolate and characterise putative OSCs, we used two commercial antibodies against the extracellular C-terminus of DDX4 for fluorescent sorting of dissociated human ovarian cells using a more refined tissue dissociation technique than previously described^5,26^. This technique involved reduced enzyme treatment to minimise cell membrane damage and antibody binding to intracellular epitopes and resulted in an increase in both the yield and viability of the cells sorted. Sufficient numbers of viable cells post-FACS can be obtained for immediate analysis of stem cell and germ-line marker RNA and protein expression which is an advance over previous studies which relied on *in vitro* expansion of isolated cells^5,20,24,30^.

Our results demonstrate that DDX4-expressing cells, that are not oocytes, can be isolated from the adult human ovary. Previous studies have isolated cells by FACS sorting using an antibody against DDX4 and have concluded that these were false positive results as they failed to demonstrate expression of DDX4 mRNA or protein either by PCR or Western blotting^30,31^. Our refined technique has consistently allowed the isolation of defined populations of DDX4-positive and negative cells using two commercially available DDX4 antibodies. However, there are cells within the positive population where DDX4 expression cannot be detected by immunostaining and these may indeed represent false positives. Within the population of cells collected from the positive gate the expression of DDX4 protein has been confirmed using the same two antibodies by Western blotting and immunocytochemistry. Gene expression of DDX4 has been confirmed by RT-PCR. Our results clearly show that a significant proportion of the positive cells isolated by FACS sorting express DDX4 and the negative cells do not. That other groups have been unable to detect DDX4 in their sorted samples highlights differences in technical approaches particularly the sensitivity of cells to the dissociation procedure, the necessity to analyse freshly isolated cells and the importance of primer design.

Previously reported DDX4 primer sequences^5,27^ bind to different regions of *DDX4*, with one set^27^ binding to the glycine rich region of the gene and the other set being complimentary to the start of the C-terminus of *DDX4*. One explanation for other researchers failing to detect *DDX4* gene expression^30,31^ could be that the particular set of primers used did not detect the isoforms present within their sample. In our P2 sample, the 5′ set of primers detected *DDX4* expression but the 3′ set of primers failed to detect any expression, suggesting that that particular isoform was lacking that region of the C-terminus of DDX4. The primers used by White *et al*.^5^, did not give positive results for any of our samples but by designing terminus specific primer sequences (designed to include known isoform sequences of human *DDX4)* different isoforms were identified.

By combining DDX4 detection with Aldefluor labelling we sought to refine the sorting process of putative OSCs. This non-fluorescent substrate for aldehyde dehydrogenase 1 diffuses into live cells and is formulated so that the fluorescent product is retained only by cells with an intact membrane, while permeabilised or dead cells appear non-fluorescent. High levels of Aldefluor staining have been utilized to identify, evaluate and isolate stem and progenitor cells^32^.

Using this rigorous sorting strategy in conjunction with molecular analysis using terminus specific primer sequences (designed to include the known isoform sequences of the human *DDX4*) allowed refinement of characterisation of DDX4-positive cells on the basis of 5′ and 3′ mRNA sequence detection, ALDH1 activity and cell size.

It is clear that cells sorted on the basis of DDX4 are not homogeneous. Three sub-populations of DDX4-positive cells were identified, differing in size and expression profiles for stem cell and germ cell markers. Identification of these sub-populations could account for the lack of uniformity between results presented by investigators seeking to produce a standardised single population of putative OSCs by FACS^5,30,31^. The size of cells in the P1 and P2 populations are broadly consistent with the diameter of the cells identified by White *et al*.^5^ suggesting that these cell populations can be isolated from the adult human ovarian cortex using a putative external epitope of the C-terminus of DDX4. Similar sized cells were also identified in lineage tracing experiments as contributing to postnatal oogenesis^33^. The DDX4-positive cells represent a heterogeneous population and we have identified a sub-population expressing *DDX4* transcripts that lack the 3′ PCR priming sites, which also uniquely expressed *DAZL*. It is suggested therefore, that the *DAZL* expressing cells contain a splice variant of *DDX4*. DAZL is a major regulator of germ cell differentiation^34–36^ and is essential for progression into meiosis^37^, thus these cells may represent a more differentiated population of cells. This finding should enable the identification of a progenitor population of OSCs and characterisation of their differentiation potential.

DDX4-negative sorted cells expressed stem cell markers *LIN28* and *POU5F1* and only one of the germline markers, *PRDM1*. Whilst PRDM1 is central to the establishment of germ cell lineage, as a translational repressor it is also involved in the terminal differentiation of B cells of plasma cell lineage^38^. It is possible that DDX4-negative cells may include a population of stem cells extant in ovarian tissue arising from lymphatic vasculature independent of the germline, and there may also be stem cells for other somatic lineages^39^. This study presents gene profile data obtained by RT-PCR. Given the heterogeneous nature of the cells, single cell analysis would be required to definitively show the co-expression pattern of each of the genes analysed.

Although the potential of any of the subpopulations of the ovarian cells identified here to develop into differentiated germ cells is still unknown, a preliminary finding of this study is that a heterogeneous population of DDX4-positive cells did, in combination with FODSCs, give rise to structures which had the appearance of early follicles. The results from a small number of cell aggregations of putative OSCs with somatic cells derived from fetal ovaries at the period of development when germ cell differentiation and follicle formation is still occurring suggests that this combination of cells can form follicle like structures. Much more detailed characterisation and determination of optimal somatic cell support is required. Even so, these early observations are encouraging. The establishment of a robust methodology to obtain distinct subpopulations of DDX4-positive cells from biopsied human ovarian cortex provides an opportunity to assess thoroughly the characteristics of these cells and their developmental potential *in vivo* and *in-vitro*.

## Methods

### Tissue collection and cryopreservation

Human ovarian biopsies were obtained from 33 women aged 22–43 years (mean ± SEM 32.8 ± 0.9 years) undergoing elective Caesarean section with written informed consent and Ethics committee approval from South East Scotland Ethics Committee 01 (ref LREC 16/SS/0144). Ovarian tissue was obtained from the Simpson Centre for Reproductive Health, Little France Crescent, Edinburgh EH16 4TJ and St John’s Hospital, Howden Road West, Livingston, EH54 6PP. All experiments were performed in accordance with UKRIO code of practice for research and the University of Edinburgh School of Biological Sciences Ethics Assessment. No identifying information was included with tissue samples; patient’s age, parity and regular medication at time of biopsy retrieval was disclosed. After transport in holding medium^40^, tissue was vitrified using a modification of a previously published protocol^41^. Briefly, tissue pieces were equilibrated for 25 minutes in vitrification medium no.1 [holding medium with HSA replaced with 20% human serum, 7.5% ethylene glycol (Sigma-Aldrich Ltd, Dorset, UK) and 7.5% dimethyl sulfoxide (DMSO) (Fisher Scientific UK Ltd, Loughborough, UK)]. Tissue was transferred into vitrification medium no.2 [vitrification medium no.1 with ethylene glycol and DMSO increased to 20% and supplemented with 0.5 M sucrose] for 15 minutes prior to open freezing and storage in vapour phase of liquid nitrogen (−135 °C).

### Thawing and tissue dissociation

Tissue was thawed by submerging into pre-warmed (37 °C) holding medium with HSA replaced by 20% human serum and 1 M sucrose for 1 min then submerged in holding medium with HSA replaced by 20% human serum and 0.5 M sucrose for 5 minutes at RT. Tissue was then rinsed three times in fresh holding medium. Tissue was disrupted thoroughly by repeated snipping by fine scissors and brief mechanical dissociation (108 s) at (37 °C) using a GentleMacs dissociator (Miltenti Biotec, Surrey, UK) with 1.2 U/ml collagenase I/II (both Roche Diagnostics Ltd, Redhill, UK). Enzymes were then inhibited using 2% NGS and the cell suspension passed through a series of filters of decreasing pore size (100-30 µm) (Partec UK Ltd, Kent, UK). Filtrate inspection under light microscopy allowed removal of any remaining follicles or oocytes. Cell viability was determined by the Trypan blue exclusion test, and expressed as a percentage of total cell number.

### Immunocytochemistry and FACS

The dissociated filtrate was centrifuged at 300 × *g* for 5 minutes, the supernatant removed and discarded and the cell pellet re-suspended in HBSS with 2% HSA and 2% NGS (blocking solution). For HEK 293 T cells, transfected with pFLAG-DDX4-myc (Supplementary Information and Supplementary Figure S1), scrapers were used to remove adherent cells from 6 well plates. Samples were then centrifuged as above. Aliquots were used for unstained and secondary antibody-only controls. Remaining cell suspension was centrifuged at 300 × *g* for 5 minutes, the supernatant discarded and the cell pellet re-suspended in primary antibody (1:100, rabbit anti-DDX4 antibody ab13840; Abcam, Cambridge, UK; 1:100, rabbit anti-DDX4 antibody LS-C97782, Lifespan Biosciences, Nottingham, UK) for 50 minutes on ice and washed before staining with secondary antibody, donkey anti-rabbit IgG antibody conjugated to cyanine 3 (Cy3) or goat anti-rabbit IgG antibody conjugated to cyanine 2 (Cy2) (Stratech Scientific Ltd, Suffolk, UK) at a concentration of 1:250 for 30 minutes on ice. Controls i.e. unstained cells and secondary antibody-only cells were used to define the gating strategy for DDX4 cell sorting. Forward Scatter (FSC) versus side scatter (SSC) profiles were used to identify intact cells, verified by additional DAPI staining of control cell suspensions. Secondary antibody-only controls were utilised to determine DDX4 discrimination. A minimum of 100,000 intact cells were recorded to allow subsequent gating. DDX4-positive and negative cells, for both human ovarian cells and transfected HEK 293 T cells, were sorted using an Aria IIu cytometer equipped with a 488 nm laser, running BD FACSDiVa v6 software (BD Biosciences, Oxford, UK). Cells were sorted on high purity setting at no more than 5000 events/s using a 70 µm nozzle at 55 psi with a 90 Hz droplet stream. Where collection allowed, samples of sorted cells were evaluated for sort purity by reacquisition. DDX4-positive and DDX4-negative cells were collected into Trizol reagent (Life Technologies, Paisley, UK) for RT-PCR for gene expression analysis or into holding medium for protein detection by immunoblotting: ≥ 5 × 10^3^ cells of each population were collected for gene expression analysis and ≥ 1 × 10^6^ cells for protein expression. Micro-beads were used to estimate the diameter of sorted cell populations.

### FACS with ALDH1 activity

FACS was refined to sort the cell sample by dual detection of DDX4 and ALDH1 activity thus identifying vital cell populations with both stem and/or germline character. Following incubation with the secondary antibody, activated Aldefluor (Stemcell Technologies, Cambridge, UK) reagent was added to the sample cell suspension at a concentration of 5 µl/ml. A quenched Aldefluor control was established by adding diethylaminobenzaldehyde (DEAB) to an aliquot of the Aldefluor cell suspension at a concentration of 10 µl/ml to inhibit ALDH1 activity. Sample and control were incubated at 37°C for 15–30 mins, before centrifuging for 5 min at 250 × *g*. Supernatant was removed and pelleted cells re-suspended in Aldefluor assay buffer to prevent efflux. Sample and control were sorted using an Aria IIu cytometer as described above. Cell populations sorted from sample aliquots using Aldefluor were collected in Trizol reagent for RT-PCR. The diameter of cells collected was estimated by running micro-beads of a known size. Antibody specificity was tested using dissociated rodent skeletal muscle, incubated with primary antibody (ab13840; Abcam, Cambridge, UK) with appropriate controls as described earlier for human cells. Rodent cell samples were sorted as described above and cells collected for gene expression analysis.

#### RNA extraction and RT-PCR (reverse transcription polymerase chain reaction)

Total RNA was extracted from freshly isolated DDX4-positive and DDX4-negative cells with and without Aldefluor. Cells were lysed in Trizol reagent prior to centrifuging at 12,000 × *g* for 10 mins at 4 °C followed by 5 mins incubation at room temperature. Chloroform was added to the supernatant for phase separation prior to further centrifugation (12,000 × *g* for 15 mins at 4 °C). RNA was precipitated with isopropanol (10 mins at RT) and recovered by centrifugation (12,000 × g for 10 mins at 4 °C). The RNA pellet was washed in 75% ethanol, vortexed, centrifuged (7500 × g for 5 mins at 4 °C), air dried and dissolved in RNase-free water. RNA was quantified using a nanodrop; ≥ 150 ng/µl of RNA was extracted in any experiment. cDNA was generated using Omniscript Reverse Transcription Kit (Qiagen, Manchester, UK) according to the manufacturer’s instructions; ≤ 2 µg of RNA was used per cDNA synthesis. PCR was performed using Taq DNA Polymerase (Qiagen, Manchester, UK) according to the manufacturer’s instructions. Primers were designed using MacVector7.2 (Table 1). For controls, RNA was extracted from cell suspensions of dissociated human ovarian tissue and rodent skeletal muscle using the protocols described above. A positive control was established using gene fragment template DNA. Housekeeping control, GAPDH, was expressed highly in all sorted cells. Negative controls were established by running replicate reactions omitting reverse transcriptase.

#### Protein detection by Western blot analysis

Freshly sorted cells were centrifuged at 300 × *g* for 5 minutes, re-suspended in RIPA extraction buffer (Fisher Scientific, Loughborough, UK) supplemented with 1% Halt Protease and Phosphatase Inhibitor Cocktail (PI) (Thermo Scientific, Loughborough, UK) and 1% phenylmethanesulfonyl fluoride (PMSF) (Sigma-Aldrich Ltd, Dorset, UK) and homogenised using a GentleMacs dissociator. The sample was centrifuged at 3400 × *g* for 5 minutes and protein was purified using Vivaspin tubes (Sartorius Mechatronics Ltd, Epsom, UK) with 50 kDa filters. Protein concentration was determined using Coomassie-Plus Reagent (Thermo Scientific Pierce, Northumberland, UK). Protein samples were denatured at 100 °C for 30 minutes, 50 µg was loaded onto 4–20% gradient gels (Life Technologies, Paisley, UK) in Tris-glycine/SDS running buffer (25 mM Tris-HCl, 52 mM glycine, 0.1% SDS) and run at 125 V for 90 minutes. Proteins were transferred to nitrocellulose membranes (Amersham Pharmacia), blocked overnight at 4 °C in TBS-T supplemented with 2.5% non-fat milk powder before probing with primary antibodies (DDX4, ab13840 (1:100) and LS-C997782 (1:200) and Histone H3, ab39655 (1:100),and secondary antibody (AffiniPure goat anti-rabbit IgG, Jackson Immuno Research, Suffolk, UK), and visualised using enhanced chemiluminescence (GE Healthcare, Life Sciences, Amersham, UK). Controls were established using protein extracted from cell suspensions of rodent testes and skeletal muscle.

#### *In-vitro* development of FACS sorted cells

A preliminary experiment was set up to assess the potential of FACS- sorted cells to undergo development *in-vitro* by combining them with Fetal Ovarian Derived Somatic Cells (FODSCs). FODSCs were derived from a 16 week gestation morphologically normal fetus following medical termination of pregnancy. Maternal informed consent was obtained and approval granted for the study by local Ethics Committee. Derivation of FODSCs, culture, expansion and thawing were performed as previously described^42^. For cell aggregation, thawed FODSCs were either cultured alone (n = 5) or combined with isolated DDX4-positive cells (P1 and P2 combined 6–8 microns in diameter) (n = 14) at a ratio of 5:1 and pipetted thoroughly to ensure the cell types interspersed. Sufficient OSC culture medium^26^ was added to give a final concentration of 1.5 × 10^5^ cells/mL. The cell suspension was seeded into wells (200 µL/well; 30,000 cells per aggregation) of a round bottom, ultra-low attachment culture plate (Costar Corning Inc, Kennebunk, Maine, USA) and centrifuged at 1200 g for 5 minutes to pellet the cells. Thereafter the cell aggregations were incubated at 37 °C with 5% CO_2_; half the medium was replaced every 2 days. After 10 days *in-vitro*, aggregates were fixed in either neutral buffered formalin (NBF) for 30 minutes, for haematoxylin and eosin staining, or microtubule stabilising buffer supplemented with 3.7% (v/v) formaldehyde, 0.1% (v/v) Triton X-100, 1 mM taxol, 0.01% (w/v) aprotinin, 1 mM dithiothreitol and 50% (v/v) deuterium oxide as described previously^43^, for 30 minutes at 37 °C, preceding fluorescent immunostaining for DDX4.

#### Histological analysis of DDX4-positive/FODSC aggregates

NBF-fixed aggregates were dehydrated in increasing concentrations of ethanol (70, 90 and 100%) then placed in cedar wood oil for 24 h (BDH Laboratory Supplies, Poole, UK) before clearing with toluene (Fisher Scientific UK, Loughborough, Ltd) for 30 mins. Aggregates were individually embedded in paraffin wax at 60 °C for 4 h with hourly wax changes, cut into sections of 6 µm, mounted onto slides, and left to dry overnight prior to staining with haematoxylin and eosin.

### Immunostaining of DDX4-positive sorted cells and aggregates

For microscopic observation, 13 mm glass coverslips were inserted into individual wells of 12 well multiwell plates (Sigma), and incubated in poly-L-lysine (Sigma), to promote cell adhesion, for 1 hour (at 37°C). Post FACS, freshly sorted cells were centrifuged at 300 × *g* for 5 minutes and resuspended in either OSC culture medium^26^, (for sorted human ovarian cells) or Dulbecco’s Modified Eagle’s Medium (DMEM) supplemented with 10% fetal calf serum, glutamine (4.5 g/L) and 1% antibiotic-antimyotic (Gibco, Life technologies)(for transfected HEK 293 T cells), before being seeded onto the poly-l-lysine coated coverslips and incubated for 24 hours (37 °C and 5% CO_2_). Immunocytochemistry was performed as previously described. In brief, cells were washed with 1 × Phosphate Buffered Saline (PBS) and fixed using NBF. Blocking solution was added to the cells for 20 minutes, before being incubated for an hour in both primary (1:100, anti-DDX4, abcam ab13840 or LS-C97782) and secondary antibodies (1:250, goat anti-rabbit IgG antibody conjugated to cyanine 2 (Cy2) (Stratech Scientific Ltd). DAPI was applied to the cells for 15 minutes (1 µg/ml), and mounted with ProLong Diamond antifade reagent (Thermo Fisher Scientific, Paisley, UK) to prevent photobleaching.

Immunostaining of the fixed aggregates was performed as previously described^44^. In brief, the aggregates were incubated in primary antibody (1:100, ab 13840) for one hour, 37 °C, with gentle agitation. The aggregates were washed 3 times in wash buffer (10 minutes each) before incubation in the secondary antibody (Alexa 488 goat anti-mouse IgG (1:500; Thermo Fisher Scientific) for one hour, 37 °C, with gentle agitation. Aggregates were then washed 3 times in wash buffer, incubated in DAPI (1 µg/ml) for 15 minutes and mounted with ProLong Diamond antifade reagent (Thermo Fisher Scientific).

### Equipment and settings

Confocal images were acquired using Zeiss LSM 800 AxioExaminer equipped with 3 GaAsP detectors and Zen Blue software (Carl Zeiss Jena GmbH, Germany). 2 acquisition channels were set up for sequential imaging. 488 nm laser was used for exciting the green channel with an emission window set to 490–550 nm (suitable for Alexa fluor 488). 405 nm laser was used for exciting the blue channel with an emission window set to 490–550 nm (suitable for DAPI). A variable beam splitter dichroics (VSDs) was used divert the respective spectral parts of the fluorescence emission light to the detectors. 20 × plan Apochromat/NA 0.8 objective (providing a lateral resolution of 305 nm at 488 nm wavelength and 253 nm at 405 nm wavelength) was used to capture the images with 1024 × 1024 pixels (with pixel size 0.623 µm for Figs 3bi,iii,v,vii and 5a and 0.125 µm pixel size for Fig. 3bii,iv,vi and viii. 2 times frame averaging was used to improve the image quality and the pixel dwell time was set to 0.746 µsec.

The bit depth of the images were 8 bit and a linear LUT was used for both channels (Figs 3b and 5a) and it covers the full range of data. Image processing was done using ImageJ^45^ and the image brightness was adjusted keeping gamma value one.

#### Histology images

Bright field colour images were acquired using a Leica DMRE (Leica Microsystems, Wetzlar and Mannheim, Germany) upright microscope with Retiga 2000R monochrome camera (Photometrics, Arizona, USA). A liquid crystal RGB Colour Filter Module (Model RGB-HM-S-IR, QImaging) was placed in front of the camera for colour imaging. X63 PL Apo OIL /NA 1.4 objective was used for capturing the images with camera settings: 1600 × 1200 pixels with image bit depth: RGB colour 24 bit. QCapture Pro6.0 (Photometrics, Arizona, USA) was used for image acquisition and the post processing was done using ImageJ^45^.

### Data availability

Raw data files can be obtained by reasonable request to the corresponding author.

All data generated or analysed during this study are included in this published article and its Supplementary Information files.

## Electronic supplementary material

Supplementary Information
