# Supplementary material for: Initial characterisation of adult human ovarian cell populations isolated by DDX4 expression and aldehyde dehydrogenase activity
Source: Sci Rep. 2018 May 3;8:6953. doi: 10.1038/s41598-018-25116-1 (PMC5934371; doi:10.1038/s41598-018-25116-1)

Initial characterisation of adult human ovarian cell populations isolated by DDX4 expression and aldehyde dehydrogenase activity.

Yvonne L Clarkson[1,2] Marie McLaughlin[1,2] Martin Waterfall[2], Cheryl E Dunlop[1,3], Paul A Skehel[1], Richard A Anderson[3], Evelyn E Telfer[1,2]

[1]Institute of Cell Biology and the Centre for Integrative Physiology, Hugh Robson Building, University of Edinburgh, Edinburgh, EH8 9XD, UK

[2]School of Biological Sciences, Ashworth Laboratories, the King's Buildings, University of Edinburgh, Edinburgh, EH9 3JR, UK

[3]MRC Centre for Reproductive Health, Queens Medical Research Institute, University of Edinburgh, Edinburgh, EH16 4TJ, UK

Y.L.C and M.McL have contributed equally as first authors.

Supplementary Information

Cell culture and transfections

Human embryonic kidney (HEK) 293T cells were maintained at 37°C and 5% CO_2 in T-75 flasks (Thermo Scientific) in Dulbecco's Modified Eagle's Medium (DMEM) supplemented with 10% fetal calf serum, glutamine (4.5g/L) and 1% antibiotic-antimyotic (Gibco, Life technologies). Cells were passaged at 90% confluence and media changed every 4 days. For microscopic observation, 13 mm glass coverslips were inserted into individual wells of 12 well multiwell plates (Sigma), and incubated in poly-L-lysine (Sigma), to promote cell adhesion, for 1 hour (at 37°C). Cells were pelleted (800 rpm for 2 mins) and diluted in 5ml media. 1% of the cell suspension was used to seed cells 24 hours prior to transfection. Lipofectamine 2000 (Invitrogen) was used to transfect the cells, at a ratio of 2:1 of DNA:transfection reagent, according to manufacturer's instructions. In brief, the Lipofectamine and plasmid DNA were separately diluted in 50 µl Opti-MEM reduced serum medium (Invitrogen). Tubes were vortexed briefly and incubated for 5 min at room temperature (RT). After incubation, tubes were combined and incubated for 20 min at RT, enabling the DNA-Lipofectamine complexes to form. Complexes were added to each well, already containing supplemented DMEM, and incubated at 37°C. The medium was replaced after 2 hours to remove

the Lipofectamine. After 48h incubation, cells were harvested for immunocytochemistry, Western blot and FACS experiments.

Generation of Novel Constructs

pFLAG-DDX4-myc

Differential polypeptide tags and restriction sites were added to the N and C termini of full length human DDX4. Restriction digestion followed by 3-way ligation allowed the insertion of this novel clone into the CMV-based mammalian expression vector, pDsRed2-C1 vector. This vector was fluorescently tagged (with red fluorescent protein, RFP) to allow for DDX4 protein detection and confirm transfection efficiency.

Due to the large size of full length human DDX4 (2187 bp), two gene block fragments were designed (Integrated DNA technologies). The first fragment (hereby referred to as N-DDX4) comprised of the first half of human DDX4 with the insertion of a *BglII* site and a FLAG tag sequence preceding the start codon of the DDX4 sequence. This gene block sequence was 1065 bp in length and terminated 5 bases after the endogenous *XhoI* site in the DDX4 sequence. The second gene block fragment (hereby referred to as C-DDX4, 1122 bp) initiated with an overlinking region with the first gene block fragment (refer to Supplementary Figure 1). The initial stop codon of the human DDX4 sequence was removed to allow for the introduction of a myc tag at the end of the C terminus of the DDX4 sequence. The stop codon was

then placed after the myc tag but before a *KpnI* restriction site, which had been introduced (for vector maps refer to Supplementary Figure S1).

Restriction digestion and 3-way ligation

200ng of N-DDX4 and C-DDX4 were double digested sequentially with *BglII* and *XhoI* and *KpnI* and *XhoI* respectively. 500ng of pDsRed2-C1 vector was sequentially, double digested with *BglII* and *KpnI*. The enzyme which had the lowest percentage activity in the NEB buffer was added first to allow for the longest digestion period, followed by the addition of the second enzyme 3-5 hours later. NEB buffer 3.1 was selected for optimal salt concentration. For complete digestion, the mixture (H_20, buffer, plasmid and enzyme(s)) was left overnight at 37°C. Digested products were resolved by electrophoresis on 0.8% TAE agarose gel and purified according to manufacturer's instructions for the Zymoclean™ Gel DNA Recovery Kit. Ligation was performed using T4 DNA ligase (2.5U, Invitrogen) at room temperature (RT) overnight. The ratio of N-DDX4:C-DDX4:vector was 1.5:1.5:1.

2µl of the ligation mix was added to 25µl of One Shot TOP10 Chemically Competent *Escherichia coli (E.coli)* cells (Invitrogen). Heat-shock transformation was performed (20 minutes on ice, 40 seconds at 42 °C, 2 minutes on ice) and competent cells were recovered in 200µl SOC media (Clontech) and incubated at 37°C for 40mins with shaking. Aliquots of the transformation product were plated onto LB

agar plate supplemented with 50µg/ml kanamycin. Plates were left in the 37°C incubator overnight. 3-6 single bacterial colonies were picked and inoculated into 2mL of LB medium supplied with 0.1% concentration kanamycin. Cultures were left to grow overnight at 37 °C with shaking. The QIAprep Spin Miniprep Kit (QIAGEN) was used to extract plasmid DNA according to manufacturer's protocol.

Removal of RFP

Removal of RFP from the vector was achieved with restriction enzymes *AgeI* and *BglII*, followed by re-ligation of the plasmid using T4 ligase. The resulting DNA was cut again with *AgeI* and *BglII* again to remove any re-ligated insert and used for bacterial transformations.

The DNA was also sequenced using 3 different primers. The first primer was located within the CMV promoter (ACGTCAATGGGAGTTTG), the second within DDX4 (5' GGCTGCTTCATCTACAAGG 3') and the third within the SV40 terminator (CCTCTACAAATGTGGTATGG). Sequencing results confirmed that full length DDX4 was successfully cloned into the pDsRed2-C1 vector.

Supplementary Figure S1: Schematic representation of the novel pFLAG-DDX4-myc construct

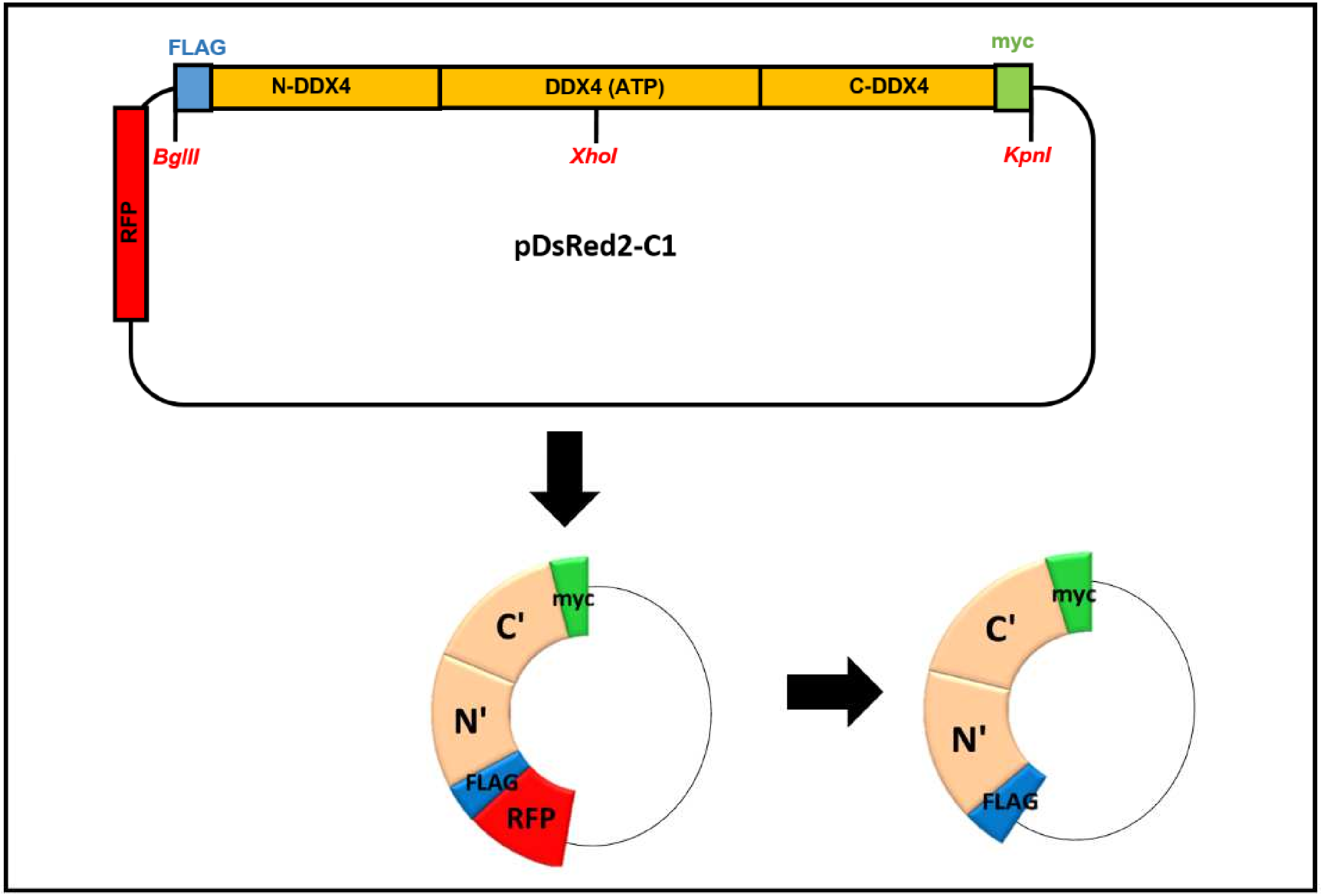

Supplementary Figure S2 Whole blot scans for Figure 2a

(i) DDX4**, 239 bp

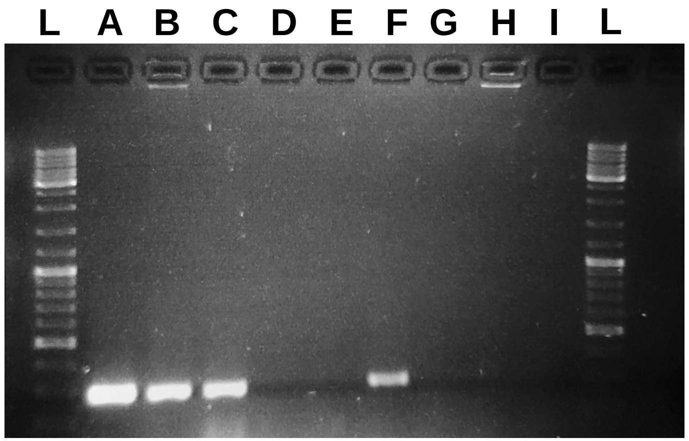

(ii) DDX4**, 239 bp

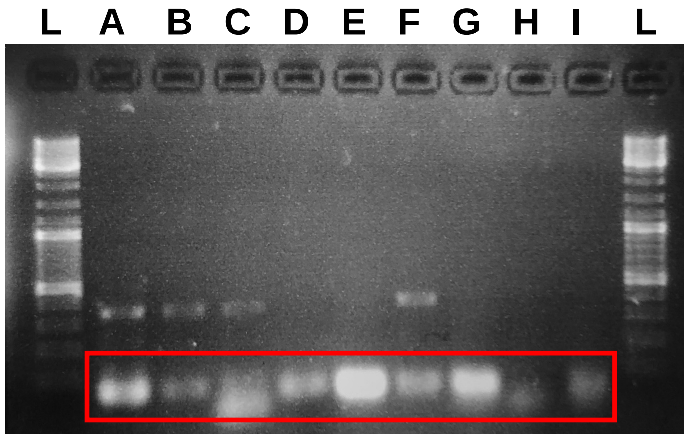

(iii) GAPDH, 227 bp

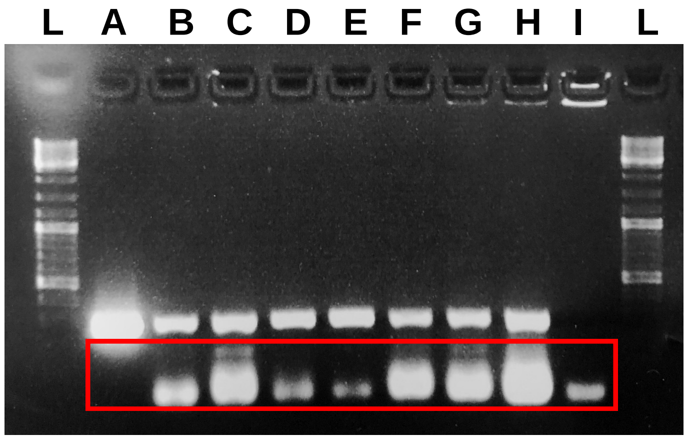

L - Ladder
A - Positive control, DNA template
B - Human adult ovary
C - DDX4-positive sorted cells
D - DDX4-negative sorted cells
E – Untransfected HEK 293T cells
E - DDX4-positive HEK 293T cells
F - DDX4-negative HEK 293T cells
G - Rodent skeletal muscle
H - No RT
Primer dimers

Supplementary Figure S3 Whole blot scans for Figure 2b

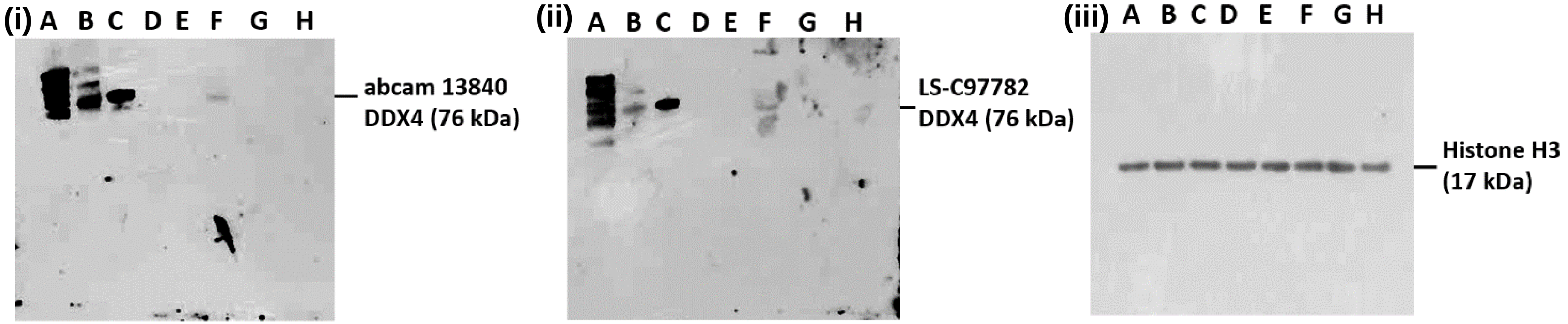

A - Rodent testes
B - Human adult ovary
C - DDX4-positive sorted cells
D - DDX4-negative sorted cells
E - Untransfected HEK 293T cells
F - DDX4-positive HEK 293T cells
G - DDX4-negative HEK 293T cells
H - Rodent skeletal muscle

Supplementary Figure S4

Telfer OSC Template Control 1

5' GAG GAC GAG ATT TGA TGG CTT GTA TAT GGG GGA ACC CAG TTG TTG TTG CTG TTG GAC AAG TGG GTG AAG AGA GGC GGC TAT CGA GAT GGA CGA AGC TGG ACA AGG AGA AGA CAT CAA AGC TCT GCA GAA AGA ACC ACA TCC CAG CCC TTG TTC AAT GTC GTC TGG TCC CTG TTC CGG GCA TCT GTA AGT GGT TCT CCT GCG GCA TTC ACG AAA CCA TGT ACG TTG CTA TCC AGG CCT CAC CCT GGG GGT TCT CTG GGT TCT ATT TGG GAA GGT ATT CAG CGT GTG GTC CCT GTT CAA CAA TTC TGC TCA TCG TCA TCC CAG AAG TCA GCC AAG GGT CTG CAG AAG TGG CAG TGG AAG TGA ACG TGG AAA GTG CCC AGT TCT TGT TGC GAT CGC ATG CTG GAT ATG CGT CAG ACC CTT ATG TTC AGT GCC GTC CAC CGC AAA TGC TTC TAT CGT GCG TGA CAT TAA GGA GCG **3'**

Telfer Template Control 2

5' TGC TGG AAG GAG TTG GAG TTG ACT TTC ACA GGT GGC ATT CCC TCC CCC TCC CCC AAA TGG AAA ATC TGG AGC AAC CAG ACC CAG AAC ATC AAG GAC TTG AGG TTT ATT CCT AAA CAT GAC CGG CTA CAA GAC CCT ATC ACG CCC AGC ACA GTC TTA TGG CAC ACC AGA GAA CCA GGC TAC AAG GAC CAG CCT CCT TCA ACC TTT AGA TCT CCA CTC TCT GGC TGA AGG TGA GGA TGC CAT TGA AGA GAA GAC CAG CAG TCC TCA GGG AAA TCG AAG AAT GTG GTC AGG TTT GAG GTT GGA CGG ATG GCG TGG TTA GGT TTG CAA ATG CCA GAC GTG CAA CAG AAG GCA AAA TCA TGC CAA ACA CCA TCT CCT TCT CGG CAT CAA TCC ATC GAT GAC GCT TCC CAA CAC AAT GGG ACG GTG GAG GCA GCA AGA GAT AGC AAG TGT TCG CAG TGC CAA GAC CAT CCT GTG TCG TGG CTC AAC TCA AGA TGT TAC TCG GAC ACT GGC TGA ATC CTT CCT GAC TTC AAT TAT GAA CGT CAG AAC CAT ACC AAT GCA AGG TGT GCC CTG ACT ACA TTG CTG CTT TCC TGC GGA GGT GGA AAA GCA ACA TTC ACG CAG AGG TCA GGA AAC TGT CAG TCT ATG ACC AGC AGA TGA TGG ACC CTG ACT TTG TTC CAG CAG TGG GAG ACA CCA AGC CAC TTC ACG GTA TGA CTT TCT CTC TGC GTC TGA TGG ACC ATT CAG GAC CAC AGT GTT CTC GAG TTC ACG CAG TTT GTG CAC TAA TGA CGT GGA TGT GCA GAA GGA GGC ATT GAC AAC AGG GTT CAT CTC AAC TGA CTG GGC AGT AGG GTG TGA GCA TGG ATT TGT **3'**

Telfer Lab Template Control 3

5’ AGT CAG CCA AGG GTC TGG AAT CGC TAC AAG TTC TAC CTG GCT TTC GAT CTT TGC CGC ACG AGT CTA CTG CTT GAG GCT CTG TCA TCA GTT TCT GGG AAG GCT TTA CCA ACC TGT CTC GAA AGA GAA AGC GAA CCA GTA TCG TGT CGT CTG GTC CCT GTT CAA CGC TCT TGG AGA TTT TCG CTT TGG TCA GCA AGC AGG AGT ATG ACG AGA GTC AGA AGC AGA AGG AGG AGA AAG AGA GCA AAT CCA TCC CCA CAC GGT GAT GCT GGT GCT GAG TAT GTG TCG GAC ATC TTC GTG CCT TAC GAA GGT GAA GGT CGG AGT CAA CGC ACA CCA TCA CCC TTT CTT CCC CAT CTC CTT GAC TGC CGC ATC ACT CCT CCA CCA CAG TTT CAG AAT GGA CCA ACA AAC AAG GAG CCT AAG GCA TCT TGG GGT AAA AGC CCC GCA TTC AAA CTG AGG TGC CTG CCT CAC ACG CTT TTC TAC AAT GGC GAT ACC AGA AAG GGC AAG AGC AGG AAG TCC CTT GCC ATC CTA AAG ACT GCG GCT TTT CTC CTA CCA GAC TGT CGC TGT AAA GAT GCT CAA CTT TGG CAT CGT GGA GGG ACT TGA GAA CTC GCA GCA CCT GGA TTG AAA TCC CAT CAC CAT CTT CCA **3’**

Supplementary Figure S5.1 Whole blot scans for Figure 4

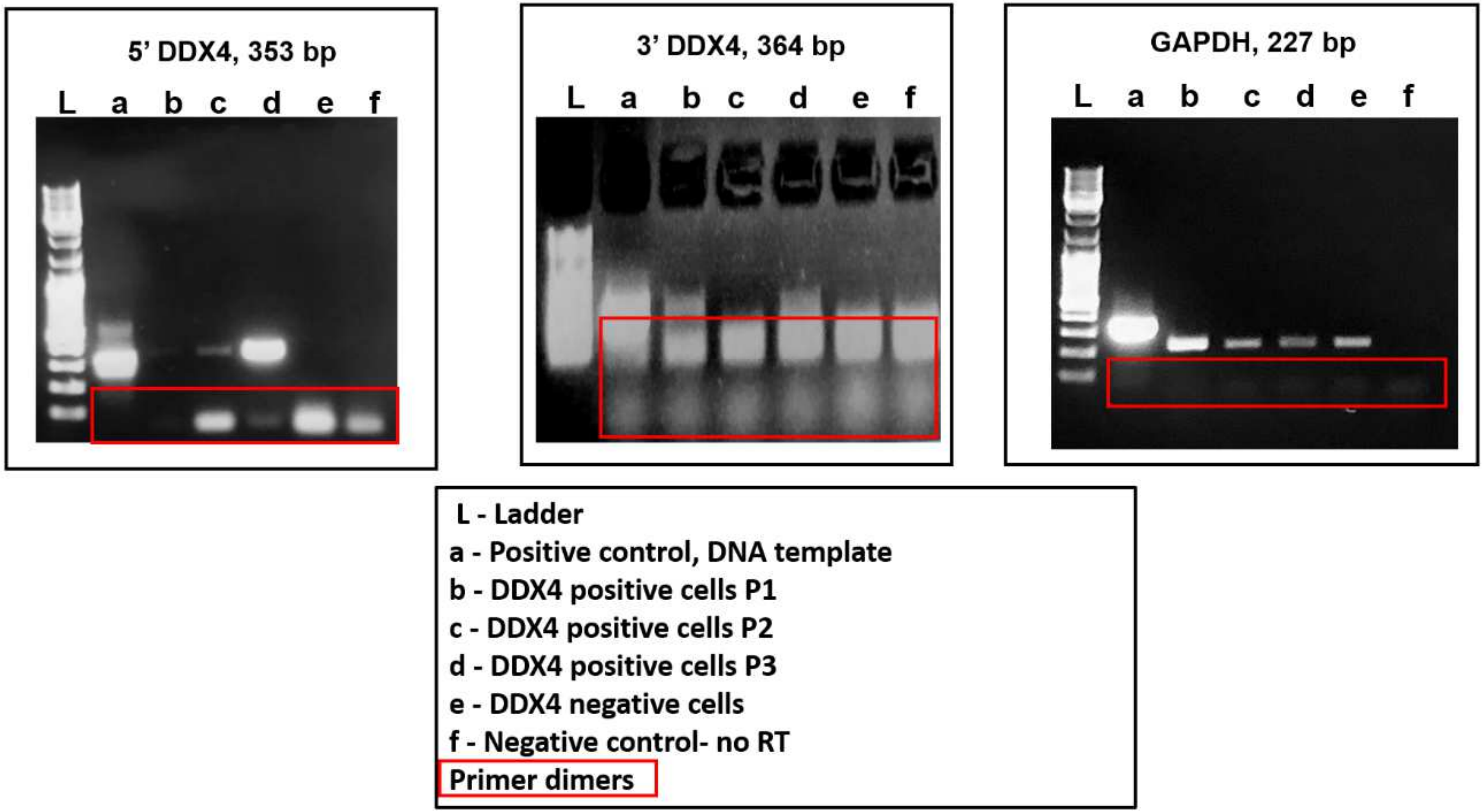

Supplementary Figure S5.2 Whole blot scans for Figure 4

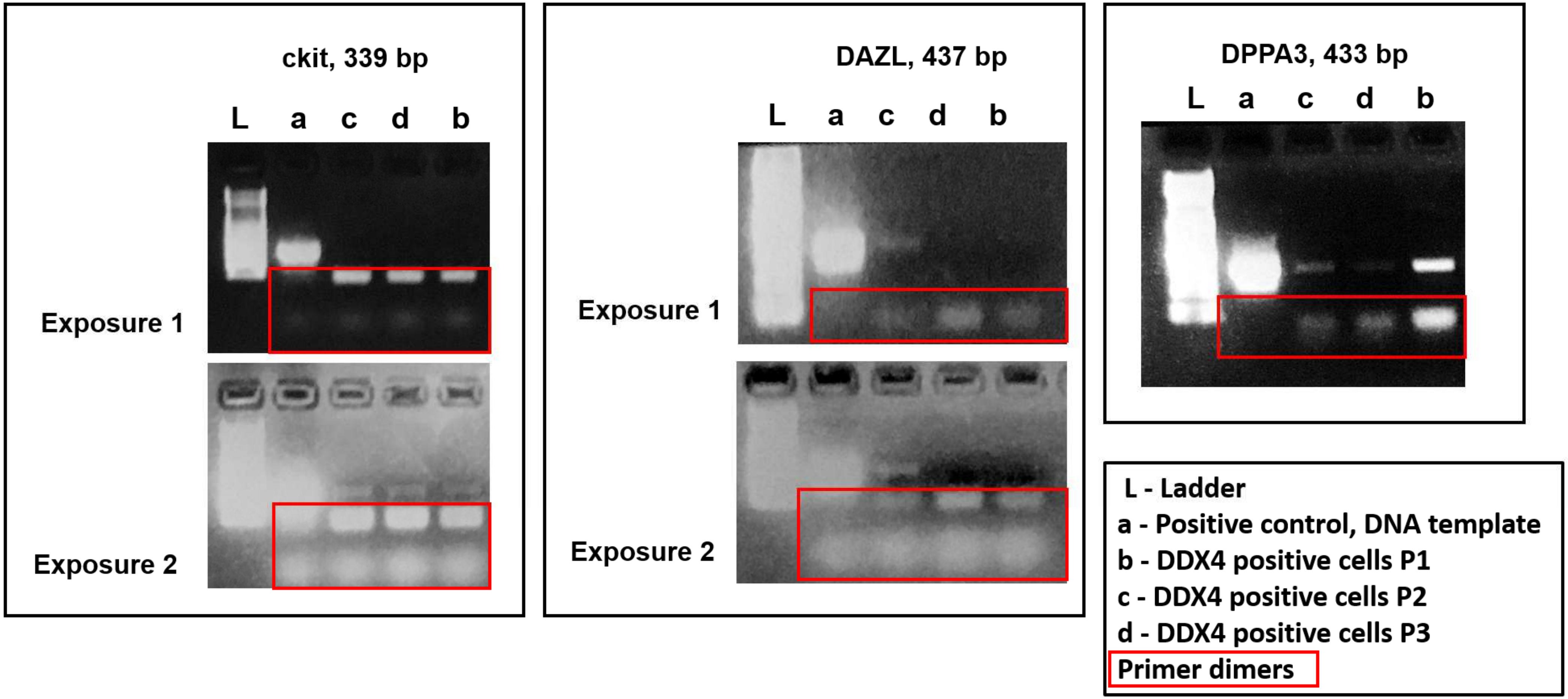

Supplementary Figure S5.3 Whole blot scans for Figure 4

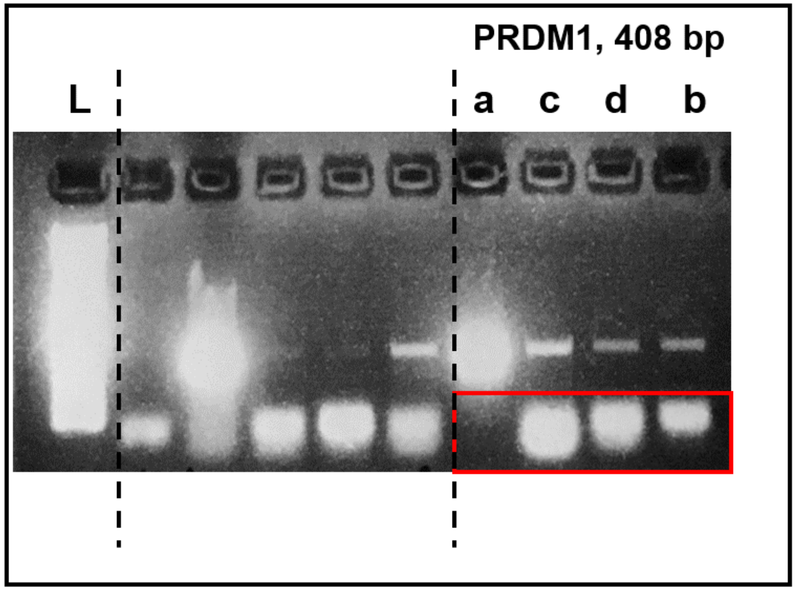

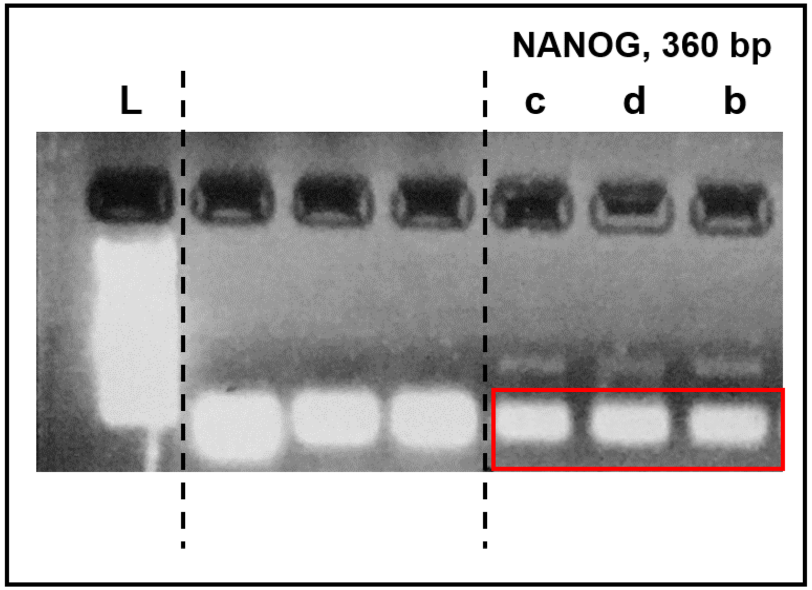

L - Ladder
a - Positive control, DNA template
b - DDX4 positive cells P1
c - DDX4 positive cells P2
d - DDX4 positive cells P3
Primer dimers
– – Additional samples not required for this paper

Supplementary Figure S5.4 Whole blot scans for Figure 4

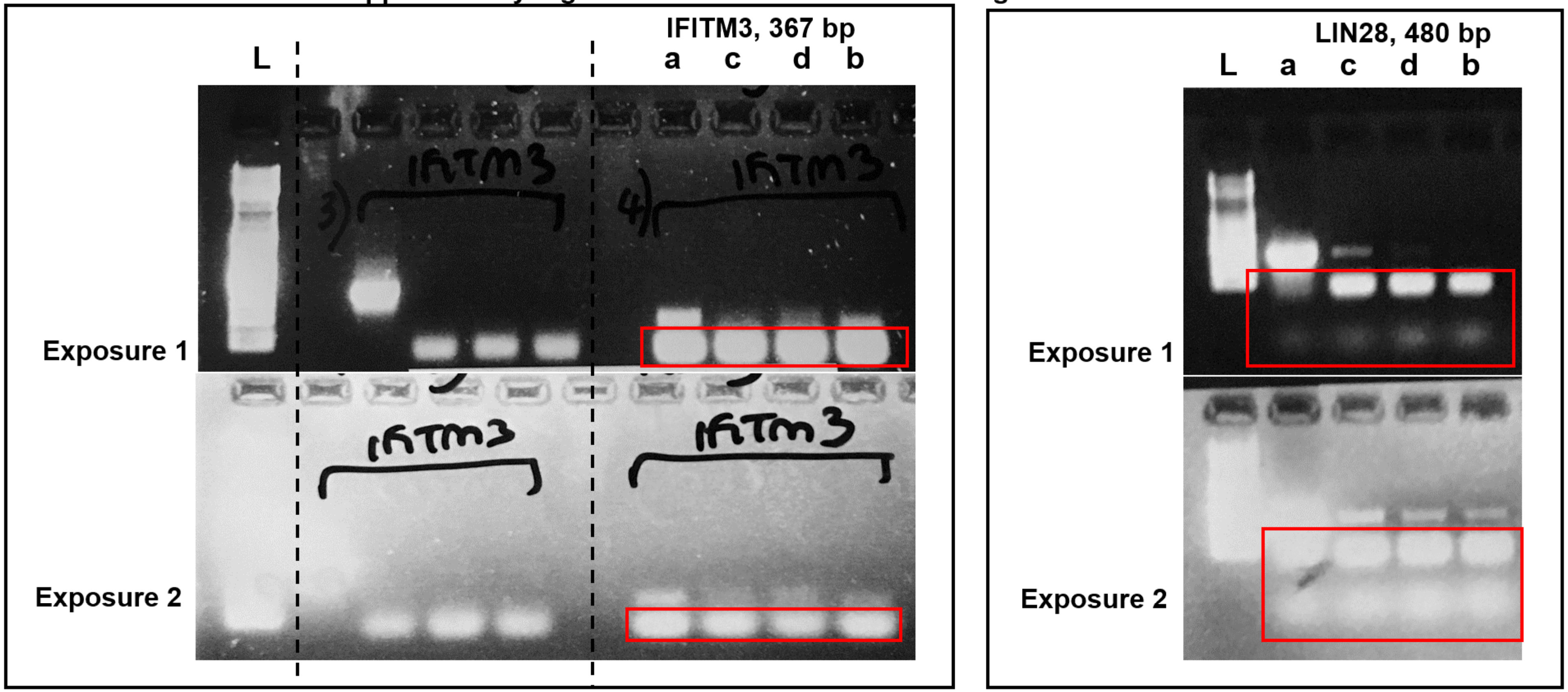

L - Ladder
a - Positive control, DNA template
b - DDX4 positive cells P1
c - DDX4 positive cells P2
d - DDX4 positive cells P3
Primer dimers
- - · Additional samples not required for this paper

Supplementary Figure S5.5 Whole blot scans for Figure 4

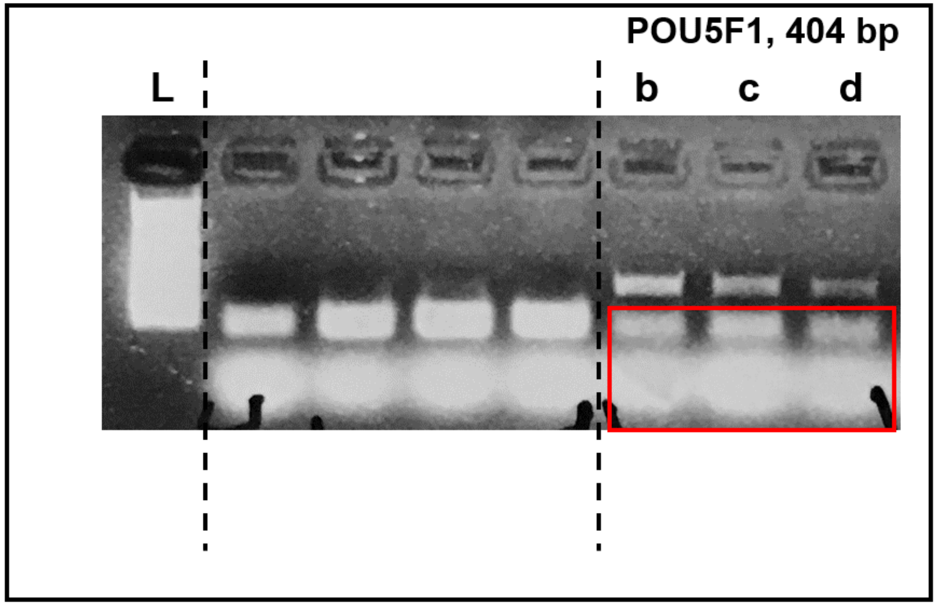

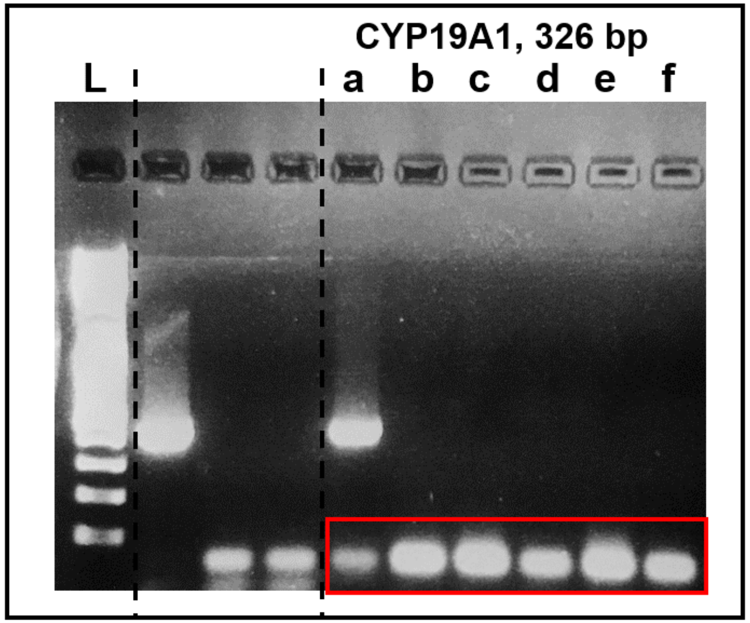

L - Ladder
a - Positive control, DNA template
b - DDX4 positive cells P1
c - DDX4 positive cells P2
d - DDX4 positive cells P3
e - DDX4 negative cells
f - Negative control- no RT
Primer dimers
– – Additional samples not required for this paper

Supplementary Figure S5.6 Whole blot scans for Figure 4

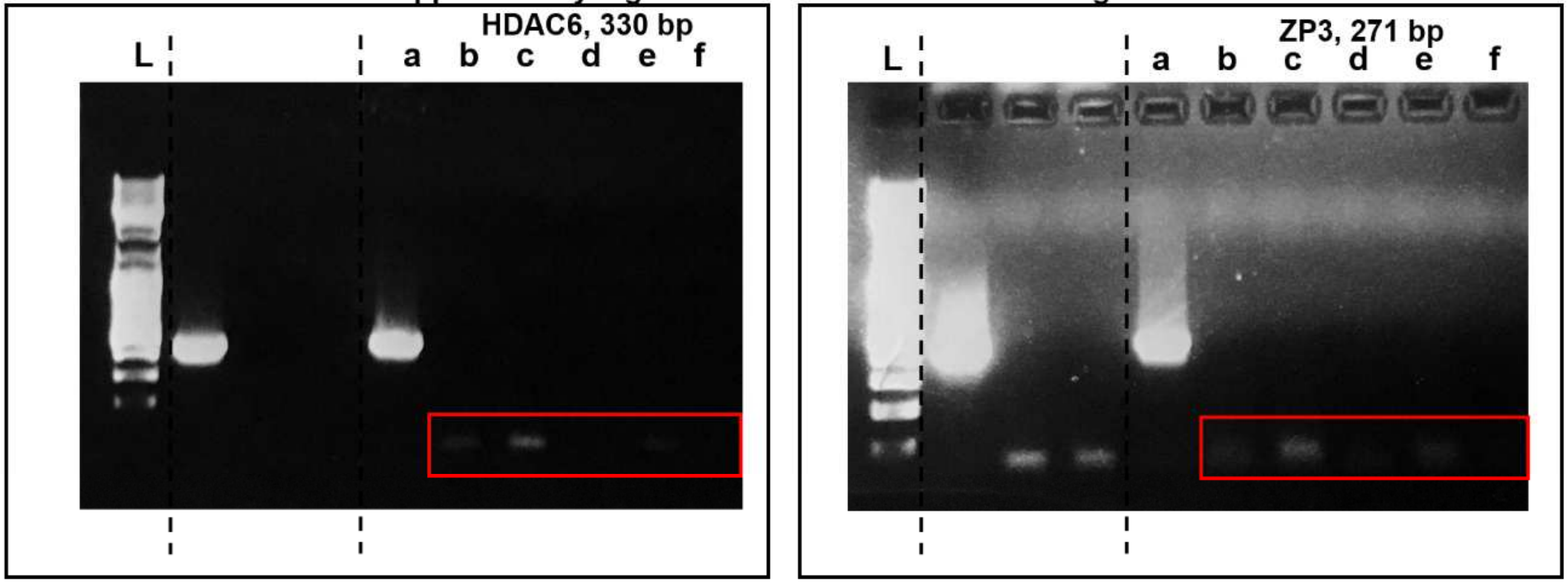

L - Ladder
a - Positive control, DNA template
b - DDX4 positive cells P1
c - DDX4 positive cells P2
d - DDX4 positive cells P3
e - DDX4 negative cells
f - Negative control- no RT
Primer dimers
– – Additional samples not required for this paper

Supplementary Figure S5.7 Whole blot scans for Figure 4

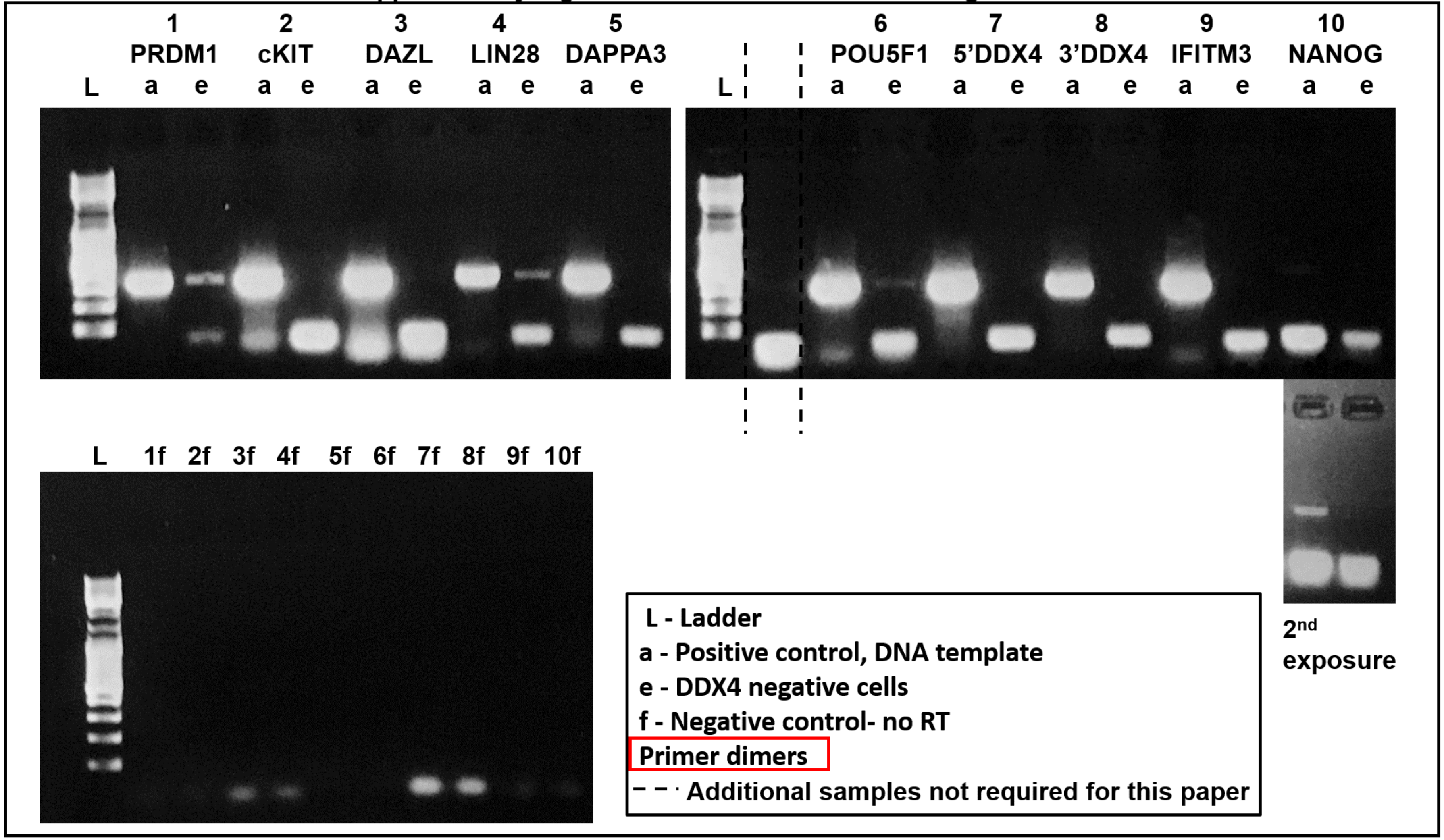

Supplement: Supplementary file 1 — Supplementary Information [file 41598_2018_25116_MOESM1_ESM.pdf]
